# Advancing transistor-based point-of-care (POC) biosensors: additive manufacturing technologies and device integration strategies for real-life sensing

**DOI:** 10.1039/d4nr04441j

**Published:** 2025-04-17

**Authors:** Xiaoao Shi, Haihui Pu, Lewis L Shi, Tong-Chuan He, Junhong Chen

**Affiliations:** a Chemical Sciences and Engineering Division, Physical Sciences and Engineering Directorate, Argonne National Laboratory, Lemont, Illinois 60439, USA.; b Pritzker School of Molecular Engineering, University of Chicago, Chicago, Illinois 60637, USA; c Molecular Oncology Laboratory, Department of Orthopaedic Surgery and Rehabilitation, Chicago, Illinois 60637, USA

## Abstract

Infectious pathogens pose a significant threat to public health and healthcare systems, making the development of a point-of-care (POC) detection platform for their early identification a key focus in recent decades. Among the numerous biosensors developed over the years, transistor-based biosensors, particularly those incorporating nanomaterials, have emerged as promising candidates for POC detection, given their unique electronic characteristics, compact size, broad dynamic range, and real-time biological detection capabilities with limits of detection (LODs) down to zeptomolar levels. However, the translation of laboratory-based biosensors into practical applications faces two primary challenges: the cost-effective and scalable fabrication of high-quality transistor sensors and functional device integration. This review is structured into two main parts. The first part examines recent advancements in additive manufacturing technologies—namely in screen printing, inkjet printing, aerosol jet printing, and digital light processing—and evaluates their applications in the mass production of transistor-based biosensors. While additive manufacturing offers significant advantages, such as high quality, cost-effectiveness, rapid prototyping, less instrument reliance, less material waste, and adaptability to diverse surfaces, challenges related to uniformity and yield remain to be addressed before these technologies can be widely adopted for large-scale production. The second part focuses on various functional integration strategies to enhance the practical applicability of these biosensors, which is essential for their successful translation from laboratory research to commercialization. Specifically, it provides a comprehensive review of current miniaturized lab-on-a-chip systems, microfluidic manipulation, simultaneous sampling and detection, wearable implementation, and integration with the Internet of Things (IoT).

## 1. Introduction

The long-lasting pandemic of COVID-19 has reiterated that infectious diseases remain one of the leading causes of death worldwide.^1,2^ According to the latest report from the World Health Organization (WHO), there have been over 775 million COVID-19 cases cumulatively and SARS-CoV-2 has caused more than 7 million deaths globally.^3^ Furthermore, the emergence of new pathogens along with the resurgence of known pathogens such as SARS-CoV-2, tuberculosis, anthrax, and influenza poses enormous challenges to public health and the economy worldwide. These highly infectious pathogens can be transmitted through air, water, food, and feces, and can enter the human body through the eyes, mouths, nostrils, ears, urogenital openings, or wounds that breach the skin barriers.^4^ Rapid and accurate detection of infectious pathogens is critical for preventing outbreaks of new epidemics and improving public health.^5,6^ In 2016, the WHO announced ASSURED criteria for point-of-care (POC) detections: *Affordable*, *Sensitive*, *Specific*, *User-friendly*, *Rapid and Robust*, *Equipment-free and Deliverable.* In May 2023, the WHO began launching global *networks for pathogen detection.*^7,8^

Compared with traditional biosensing techniques (*e.g.*, polymerase chain reaction (PCR), colony-based culture and immuno-based assays) that rely heavily on trained professionals and sophisticated laboratory instruments, miniaturized biosensors have garnered significant interest over the past few decades, owing to their suitability for POC diagnostics as well as their potential in healthcare monitoring, environmental surveillance, and food safety industries.^9–13^ POC biosensors are devices that recognize a specific type of biomolecule (DNA, antibody, antigen, bacteria, protein, virus) and generate measurable signals for real-time diagnosis through electrical, optical, biological or mechanical mechanisms. Among the most widely used POC biosensors are electronic- and optical-based systems, such as glucometers and colorimetric lateral flow assays (LFAs).^14,15^ Depending on the signal type, the optical biosensors can be further classified into fluorescence-based biosensors, surface-enhanced Raman scattering-based biosensors, surface plasmon resonance (SPR)-based biosensors and photonic crystal-based biosensors.^16–21^ However, when detecting low-concentration analytes, the signal produced by many optical biosensors falls within a range difficult to distinguish with the naked eye, requiring bulky and complex equipment to translate the optical signal into readable results. This requirement significantly hinders the transition of optical biosensors from laboratory settings to portable commercial POC devices.

**Fig. 1:**
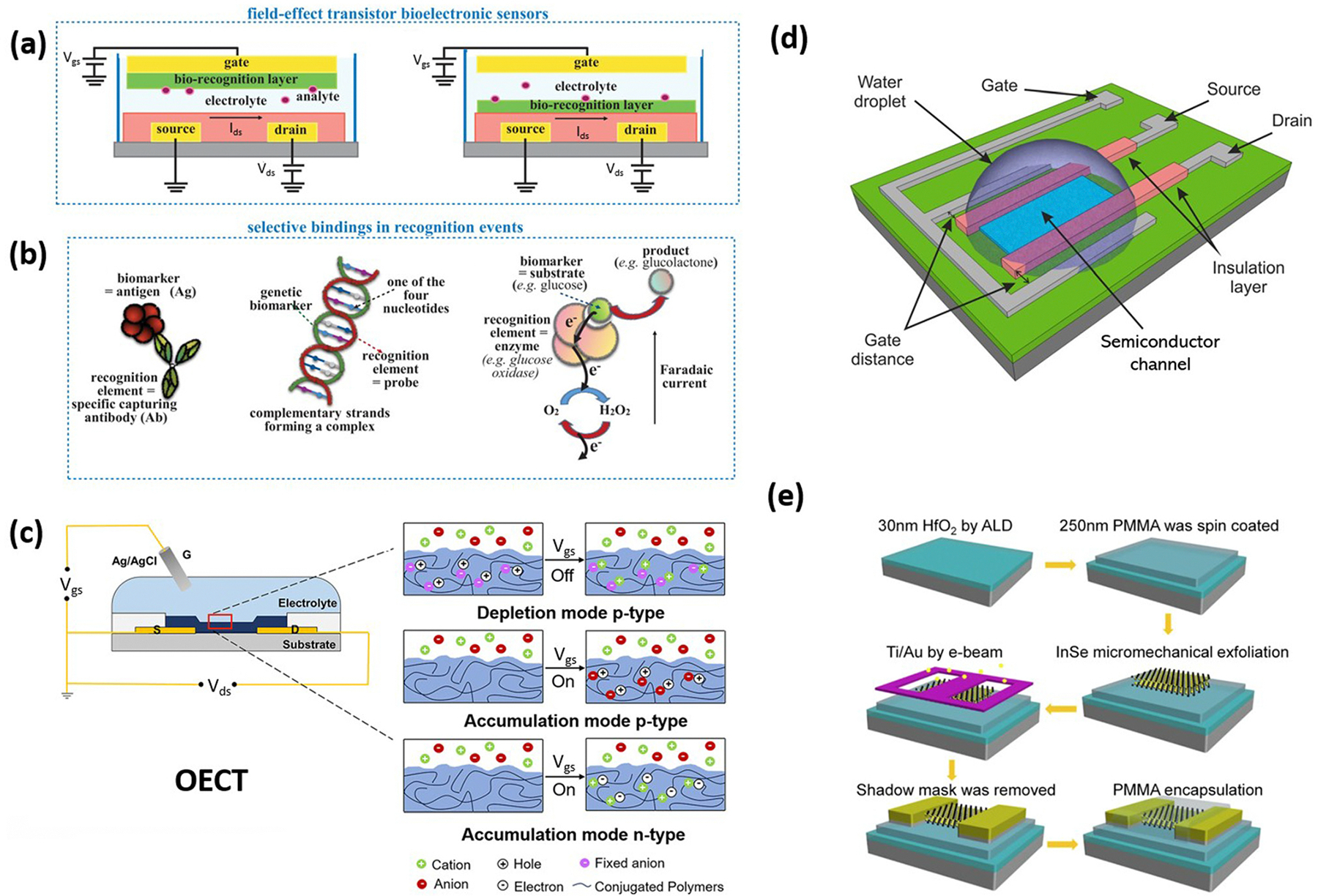
(a) Schematic of FET bioelectronics.^27^ (b) Selective binding in recognition events: specific capturing antibody binding antigen biomarker; DNA probe binding complementary DNA genetic biomarker; enzyme (glucose oxidase) electrochemical reaction with substrate (glucose).^27^ “Reproduced from ref. 27 with permission from Wiley, copyright 2019”. (c) Schematic illustration of the changes occurring in the electrolyte/polymer interface for OECTs working in depletion (top) and accumulation mode (middle for p-type and bottom for n-type).^28^ “Reproduced from ref. 28 with permission from Wiley, copyright 2021”. (d). Schematic of FET biosensor structure with all key components.^29^ “Reproduced from ref. 29 with permission from Springer Nature, Creative Commons Attribution (CC BY) license, copyright 2017”. (e). Schematic of an InSe-based FET fabrication in traditional lithography.^30^ “Reproduced from ref. 30 with permission from Springer Nature, Creative Commons Attribution (CC BY) license, copyright 2019”.

In contrast, electronic-based biosensors, which directly convert the presence and concentration of analytes into electrical signals, offer a highly promising alternative for POC due to their miniaturized form and ease of integration.^21–23^ These biosensors can be classified into four main categories based on their detection mechanisms: amperometry, potentiometry, impedance, and transistor-based sensing platforms. Among them, transistor-based biosensors, such as field-effect transistor (FET) biosensors and organic electrochemical transistor (OECT) biosensors, stand out as superior candidates due to their unique electronic characteristics, compact size, broad dynamic range, and real-time biological detection with LODs down to zeptomolar levels and, in some cases, even enabling the evaluation of single molecules or particles.^24,25^ A transistor-based biosensor is defined as a three-terminal system comprising source, drain, and gate electrodes, along with a semiconducting material channel and a highly sensitive and selective biorecognition layer (Fig. 1a & b). As target analytes bind to probes on the sensing surface, the induced electric field change results in a current/impedance change between the source and drain electrodes.^26^ This calibrated current/impedance change can be used to determine the presence and the concentration of the analytes.

Traditional FET sensors operate in two characteristic states: the “on” state and the “off” state. In the off state, carriers in the channel are depleted, causing no current flow between source and drain electrodes, while in the on state the carriers in the channel are enhanced, allowing current flow between the source and drain electrodes. The on/off current ratio, as the name suggests, is the ratio between the on-state current and off-state current and is one of the important critical performance metrics for assessing the switching ability of sensors.^26^ A high switching ability typically indicates better sensitivity of the FET-based biosensors and can be calculated through the transfer curve plot (source drain current $I_{ds}$ *vs.* gate source voltage $V_{gs}$). Switching ability (on/off ratio) is controlled by the channel material selection, insulator quality, and channel design *etc*. In ideal situations, assuming (1) there is no leakage through the insulator layer, (2) the contacts are ideal, and (3) there is no scattering of electrons by impurities in the semiconductor channel, the $I_{ds}$ can be formulated as eqn (1).

(1) $$I_{ds}=\mu C\frac{W}{L}\left[\left(V_{gs}-V_{T}\right)V_{ds}-\frac{{V_{ds}}^{2}}{2}\right]/\left(1+\frac{\mu V_{ds}}{\nu_{sat}L}\right)$$

where $I_{ds}$ is the current between the source and drain electrodes, μ is the carrier mobility, $\nu_{sat}$ is the saturation velocity of the channel material, W and L are the channel width and length, respectively, $V_{gs}$ is the voltage between the gate and source electrodes, $V_{ds}$ is the voltage between the drain and source electrodes, C is the gate capacitance C=ε/d, ε is the dielectric constant, d is the insulator thickness, and $V_{T}$ is the threshold voltage determined by the difference in the work functions of the metal and the semiconductor.^26^ Another important property of FET is the charge carrier mobility μ, with a higher μ indicating a faster responding speed and better sensitivity. Electron mobility can be expressed by eqn (2):^31^

(2) $$\mu_{FE}=\frac{L}{WCV_{ds}}\frac{dI_{ds}}{dV_{gs}}$$

Organic electrochemical transistors (OECTs) are relatively new and promising transistor-type candidates for next-generation biosensing, given their unique mechanism of operation, which involves the modulation of electrolyte concentration to regulate the channel conductivity (Fig. 1c).^24,32^ OECTs are three terminal devices similar to FET, but with semiconductor channel materials replaced by soft ion-permeable conducting polymers such as poly(3,4-ethylenedioxythiophene) polystyrene sulfonate (PEDOT:PSS). Unlike FETs, OECTs operate in an electrolyte solution, as the channel and the gate electrode must be bridged by electrolyte for proper functioning. The gate voltage controls the electrochemical doping/dedoping of the conductive polymer channel, leading to variations in source–drain current. The usage of soft conducting polymers enables OECTs to maintain electrical performance under bending cycles, making them promising candidates for wearable device applications. OECT devices have demonstrated efficacy in the detection of ions, various biomolecules, antibody–antigen binding events, and stress and strains.^33–37^

In transistor-based biosensors, the selection of conducting channel materials substantially governs the overall performance of the transistor sensor. Nanomaterials have been extensively utilized as semiconductor channels in the FET fabrication due to their large surface-to-volume ratio, favorable semiconducting properties, flexibility, and stretchability. A schematic of a typical FET biosensor structure with all the key components is illustrated in Fig. 1d. Currently, multi-step-based lithography remains the gold standard for the laboratory-scale fabrication of transistor-based biosensors. For instance, lithography followed by metal deposition is employed for the fabrication of electrodes in various FET-based biosensors,^38–40^ while soft lithography is used for fabrication of the electrolyte gates for various OECT-based biosensors.^12,37,41,42^ Chemical vapor deposition (CVD) and spin coating are commonly used to deposit various nanomaterials—graphene, MoS_2_, nanotubes, and organic materials—as a transducing channel.^43–48^ Although the traditional CVD processes provide high-quality nanomaterial layers, their application in the mass-scale fabrication of 2D nanomaterials is limited by high costs, low yields, tedious material transfer processes and usage of a high-temperature furnace.^49^ The spin coating method is noted for its good control over the thickness and uniform film quality on a small-area substrate, achieving high reproducibility through adjustments in speed, time and acceleration.^50^ However, this reproducibility tends to decrease as the substrate size increases. A schematic of an InSe FET-based biosensor, fabricated using CVD and traditional lithography, is illustrated in Fig. 1e. In contrast to these traditional methods of fabricating biosensor devices, additive manufacturing (AM) showed tremendous advantages, including ease of prototyping, reduced expensive material usage, reduced fabrication time and less instrument reliance. The capabilities of AM are continuously extended by the scientific community into a wide variety of applications, including medical diagnostics, food security, energy storage, electronic skin, artificial organs, and in-body scaffolds.^51–55^

Since most nanomaterials exhibit excellent compatibility with AM technology, numerous studies have been carried out on the integration of nanomaterials with AM for transistor biosensor fabrication, capitalizing on both the advantage of the material properties and the cost-effective fabrication techniques.^56–61^ This review will examine the fabrication of nanomaterial-based transistor-based biosensors using various AM techniques, analyzing their advantages and challenges. However, successful mass fabrication alone is insufficient for translating laboratory biosensors into commercial devices. Functional device integration is the next critical step in this transition. In the second main part, this review provides a comprehensive examination on various functional device integration strategies for transistor-based biosensors, with a focus on enhancing their practical applicability.

## 2. Additive manufacturing for transistor device fabrication

AM (or 3D printing) is defined as the process of creating physical objects through the layer-by-layer deposition of materials directly from digital design files.^62^ While printing technologies have been widely adopted in a variety of fields, such as T-shirt printing a long time ago, it was not until 1986, with the publication of Chuck Hull’s patent, that the potential of AM (3D printing) started to be recognized in sensor development.^63^ 3D printing technologies integrate the device design with device fabrication in one step, achieving high resolutions and significantly cutting down the time and effort required for device optimization.^64,65^ Although the first-generation 3D printers were prohibitively expensive for research usage, since the last decade, commercial desktop 3D printers have become available at prices ranging from $1000–$4000.^66^ In the near future, 3D printing may evolve into a mass production method, facilitating the transition of biosensors from off-site laboratories to on-site, customizable detections. Several promising techniques, such as screen printing, inkjet printing, aerosol jet printing and digital light processing, have already been used in the fabrication of transistor-based biosensors.

### Screen printing

**Fig. 2:**
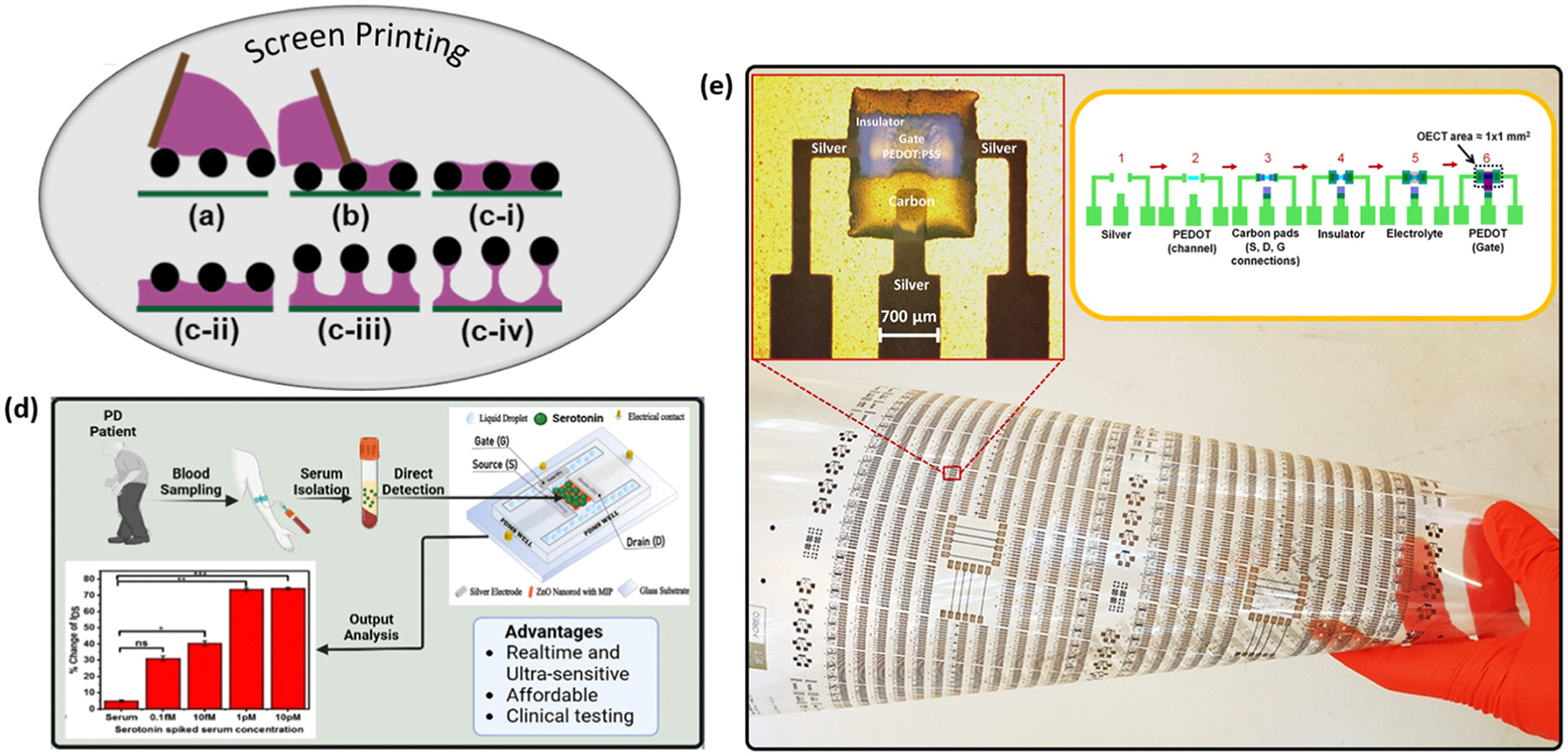
Schematic of screen printing mechanism^69^ (a) Excess fluid on the mesh after a flood stroke. (b) Squeezing forces the screen into contact with the substrate and fills the cavities with ink. (c) (i–iv) The screen separates from the substrate, and ink is pulled from mesh. “Reproduced from ref. 69 with permission from the American Chemical Society, CC-BY-NC-ND 4.0, copyright 2021”. (d). Screen-printed ZnO nanorod FET for serotonin detection in clinical samples with a molecularly imprinted polymer interface.^71^ “Reproduced from ref. 71 with permission from the American Chemical Society, copyright 2023”. (e). Sequence of six printed layers and the approximate footprint per OECT.^72^ “Reproduced from ref. 72 with permission from Springer Nature, Creative Commons Attribution (CC BY) license, copyright 2020”.

Screen printing has been utilized in the fabrication of electrodes from the early 1990s. Compared with pad printing and roll-to-roll printing, screen printing has the capability of bridging the gap between laboratory prototypes and commercialized devices.^67^ The screen printing process can be divided into three stages: in the first stage, the ink floods into the mesh and occupies the open area (Fig. 2a); in the second stage, the ink flows into contact with the substrate due to gravity (Fig. 2b); and in the final stage, the mesh is lifted off vertically and the ink forms filaments by overcoming the cohesive forces (Fig. 2c). This final stage can be further divided into 4 substages identified by Messerschmitt: adhesion of ink to the substrate, extension of the mesh, flow of the ink into filaments, and separation of the ink filaments.^68^ The filament structure that results from this transfer process is closely related to the ink viscosity, cohesive and adhesive forces, pseudoplasticity, and mesh angles.^69^ The resolution of screen printing in producing ultrathin (<70 μm) filaments is still an area of active research.^53,70^

Screen-printing enables the easy reproduction of designs on a variety of substrate surfaces, such as ductile polymers, paper, ceramics, and glass. It offers enormous versatility in electrode design and material selection while maintaining mass-producibility, cost-effectiveness, and reproducibility.^72–74^ Over the past decades, screen printing has been extensively utilized in the fabrication of electrodes, which are used in various biosensors for pathogen and biomolecular detection.^75–77^ However, using screen printing to fabricate the transducing channel in transistor-based biosensors still presents significant challenges due to limitations in printing resolution and potential damage from contact printing. As a result, the application of screen printing in FET-based biosensor fabrication remains limited, as it is not well-suited for one-step fabrication of sensors.

Only a few studies have attempted to leverage the cost-effectiveness and rapid prototyping capabilities of screen-printed electrodes for FET-based biosensor development. Sinha *et al.* fabricated molecularly imprinted polymer (MIP)-modified ZnO nanorod FET for serotonin detection, achieving a detection range from 0.1 fM to 10 pM in approximately 45 seconds (Fig. 2d).^71^ In this process, an optimized 1 : 4 diluted silver ink was used to screen-print source, drain, and gate electrodes of appropriate thickness. Subsequently, an Al- and Mg-doped ZnO thin film was deposited using the sol–gel method in a standard clean bench, followed by chemical bath deposition to grow ZnO nanorods. Careful control over the thickness of the screen-printed metal electrode layer is crucial, as it directly affects sensor performance. Excessive electrode thickness can lead to nonconformal growth of the ZnO channel layer, ultimately reducing the sensor’s On/Off current ratio.

Conversely, screen printing has been widely adopted for the fabrication of electrolyte gates in organic electrochemical transistors (OECTs). Zabihipour *et al.* explored the use of screen printing in OECT fabrication and achieved an overall yield of 99.7%, thus demonstrating the potential of this technique for large-scale production.^72^ The fabrication process of a six-layered OECT using screen printing is shown in Fig. 2e, namely the electrode, channel, connections, insulator, electrolyte and gate. In their study, up to 760 OECTs of 1 mm^2^ area were fabricated in a single batch on a PET sheet and only two devices exhibit a On/Off ratio less than 400. However, as OECT designs shrink to channel sizes as small as 150 μm × 100 μm, device performance can exceed an On/Off ratio of 100 000, but the yield decreases to below 98%, and uniformity across the entire sheet becomes worse. While such a manufacturing yield may be sufficient for standalone transistors, further improvements are necessary for printed circuitry applications. Contat-Rodrigo *et al.* also demonstrated the potential of screen printing for OECT fabrication by printing PEDOT:PSS onto a polyester film to create a flexible OECT sensor for ascorbic acid (AA) detection. The biosensor achieved a detection range from 10^−8^ M to 10^−2^ M, highlighting the feasibility of screen-printed OECT for detecting food-based AA with a concentration typically in the millimolar (mM) range.^78^

Despite its promise, several challenges remain in the application of screen-printing for transistor-based biosensor fabrication. When fabricating multilayer structures at ambient temperature, yield and reliability during mass-production *via* screen-printing are major concerns.^72^ System clogging, induced by an agglomeration of particles due to the evaporation of surfactants and solvents in the printing paste, is another common technical issue encountered during large-scale production, leading to high material waste.^79^ Several solutions have been proposed to address the system clog issue, including the dilution of particle-based ink and the use of aqueous combustible molecular precursor-based clog-free ink.^80,81^ However, optimizing screen-printing inks remains experimentally tedious, as it is closely tied to factors such as viscosity, adhesive and cohesive forces in the ink, which limits the broader application of screen-printing.

### Inkjet printing

Inkjet printing (IJP) is a non-impact printing technique that deposits nanomaterials in the form of a colloidal dispersion, droplet by droplet, through micro-meter scale nozzles. It is recognized as a material-saving deposition technique for printing functional patterns.^82^ A typical inkjet-printer consists of three main components: a motion stage, control systems (including the printing heads), and a vision system.^83^ The motion stage enables movement along the *X*–*Y* axis to print patterns, while the control system facilitates *Z*-axis movement of the nozzle heads. Additionally, the resolution is controlled by adjusting the nozzle angle, which determines the spacing between droplets. The printing nozzle, connected to an ink cartridge, ejects a small volume of droplets *via* program control. While smaller nozzles provide finer control over the droplet volume, they also increase the risk of clogging due to oversized particles or agglomeration of particles caused by over-evaporation of solvents.

The vision system typically consists of a fiducial camera and a droplet-watcher. The fiducial camera provides a white live image of the substrate, while the droplet-watcher helps visualize the ejected droplets in real time. Ink formation can occur through either thermal or piezoelectric mechanisms. In thermal nozzles, resistors act as heaters to create bubbles or negative pressure within the reservoir, allowing for precise control over the drawing and ejection of ink. In contrast, piezoelectric nozzles utilize electrical pulses to deform a piezoelectric film, thereby regulating the ejection rate. As the piezoelectric film deforms, pressure is generated, causing a specific volume of droplets, proportional to the pressure, to be ejected. New ink is then drawn into the reservoir as the film returns to its original state.

**Fig. 3:**
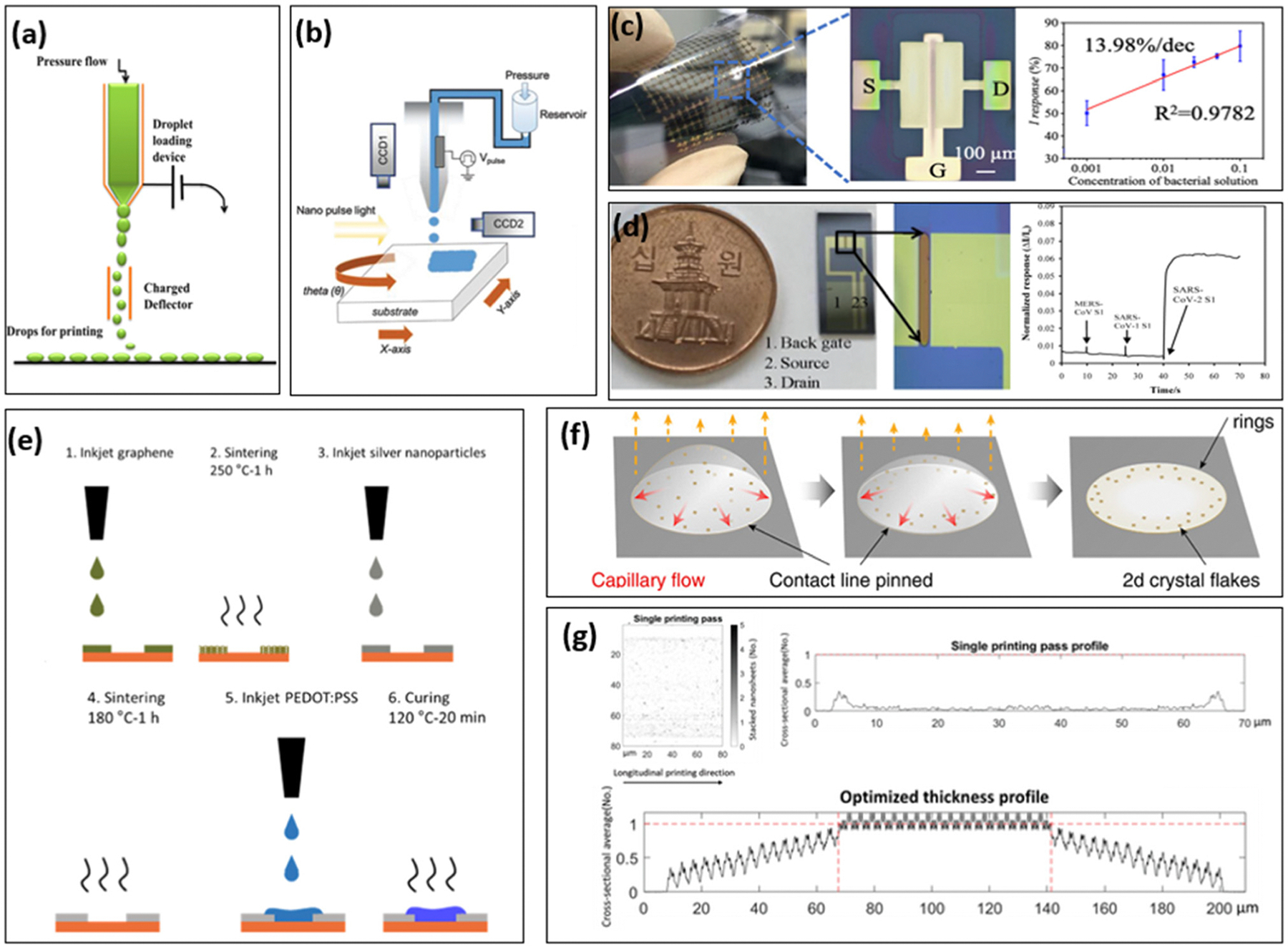
(a) Schematic diagram showing the principles of operation of a continuous inkjet (CIJ) printer.^89^ “Reproduced from ref. 89 with permission from MDPI, Creative Commons CC BY license, copyright 2017”. (b) Schematic diagram for a drop-on-demand single-nozzle inkjet printing system.^83^ “Reproduced from ref. 83 with permission from Wiley, Creative Commons CC BY license, copyright 2019”. (c) Fully printed field-effect transistor for one-drop detection of *Shewanella oneidensis* MR-1 bacteria.^58^ “Reproduced from ref. 58 with permission from the American Chemical Society, copyright 2023”. (d) Printed carbon nanotube field-effect transistor (CNT-FET) biosensor for rapid detection of SARS-CoV-2 surface spike protein S1.^90^ “Reproduced from ref. 90 with permission from Elsevier, Creative Commons CC BY license, copyright 2021”. (e) Schematics of the fabrication steps for an all-inkjet-printed graphene-gated organic electrochemical transistor enzymatic biosensor on polymeric foil.^91^ “Reproduced from ref. 91 with permission from the American Chemical Society, copyright 2022”. (f) Schematic of the coffee ring mechanism in inkjet printing.^92^ “Reproduced from ref. 92 with permission from Science Advances, Creative Commons CC BY license, copyright 2020”. (g) Thickness profile of multiple aligned inkjet printing.^93^ “Reproduced from ref. 93 with permission from Wiley, copyright 2023”.

Inkjet-printers are commonly categorized into continuous inkjet-printers and drop-on-demand inkjet-printers (Fig. 3a and b). As the name suggests, continuous inkjet printing involves the continuous ejection of ink droplets, with only uncharged droplets being selected to form patterns. Charged droplets are deflected during the passage of the deflector electrode and subsequently recycled. In contrast, a drop-on-demand inkjet printer prints out droplets of ink only on demand using either a thermal or piezoelectric mechanism. Commercially available inkjet printers, such as the Dimatix Materials Printer 2850 (Fujifilm), can achieve a line width of approximately 25 μm.^84^ A gap size down to 1 μm is achieved, using silver inks with different chemical properties that prevent mixing.^85^ Additionally, when combined with organic material inks, such as conductive polymers, photo-polymerization can be integrated into the printing process on demand, providing on-demand curing during printing. Compared with traditional semiconductor fabrication methods, such as photolithography or electron-beam lithography (EBL), inkjet printing is a promising technique for large-area fabrication of flexible electronics due to its compatibility with various substrates. Further advantages include minimal material waste, low cost, high controllability and rapid manufacturing.^51,86–88^ The inkjet-printing technology has been applied in the fabrication of FETs, organic electrochemical transistors and various other electrochemical transistors for biosensing applications over the past few decades.^83^

Owing to its compatibility with various substrates and its cost-effectiveness, inkjet-printing has been extensively employed in the fabrication of flexible or fully printed FET biosensors in recent years. Sun *et al.* developed a fully printed FET biosensor using AuNPs as electrode and carbon nanotubes as transducing channels for the detection of *Shewanella oneidensis* MR-1 bacteria (Fig. 3c). The sensor achieved an electron mobility of 70 cm^2^ V^−1^ s^−1^ and a limit of detection as low as 10 CFU per droplet (*i.e.*, 10^5^ CFU mL^−1^).^58^ Other channel materials, such as carbon nanotubes, graphene and transition metal dichalcogenides (TMDs), are also potential options for inkjet-printed FET sensors. A carbon nanotube based inkjet-printed FET has been developed for SARS-CoV-2 detection (Fig. 3d), achieving a limit of detection of 0.1 fg mL^−1^ to 5.0 pg mL^−1^.^90^ Xiang *et al.* fabricated a graphene FET on a flexible Kapton substrate for the detection of norovirus, with a detection range of 0.1 to 100 μg mL^−1^.^94^ Additionally, gold and copper-oxide inks have been used to create disposable and cost-effective non-enzymatic glucose sensors.^95^ The reported detection range is 0.5–7 mM while typical glucose levels for healthy individuals range from 4.9–6.9 mM.^96^

Since 2008, flexible organic-based electrochemical sensors have also benefited from inkjet printing technology.^97^ Inkjet printing technology is particularly well-suited for the maskless deposition of small amounts of solvent-based conductive polymers. Demuru *et al.* have demonstrated a fully inkjet-printed graphene-gated OECT for enzymatic-based biosensing of glucose on polymeric foil, achieving a linear sensing range of 30–5000 μM glucose concentration.^91^ The fabrication steps of an all-inkjet-printed graphene-gated OECT for enzymatic sensing are shown in Fig. 3e. The steps include: (1) printing layers of graphene ink on the gate electrode, (2) sintering at 250 °C for 1 h, (3) printing of silver ink for the electrical contacts, (4) sintering at 180 °C for 1 h, (5) printing of PEDOT:PSS ink for the channel, and (6) curing at 120 °C for 20 min. The gate graphene layer is 1 × 2 mm^2^ including a 1 × 1 mm^2^ overlap with gate silver contacts. All printings are done in the low-temperature environment (<40 °C) and the performance of all-printed OECT is comparable to OECTs fabricated in a cleanroom. Zea *et al.* also demonstrated a fully-printed flexible pH sensor for health care monitoring using low-cost combined polymers—polyaniline (PANI), polypyrrole (PPy) and poly (sodium 4-styrenesulfonate) (sodium PSS)—achieving a linear super-Nernstian response (81.2 ± 0.5 mV per pH unit).^98^ Molazemhosseini *et al.* developed an electrolyte-gated FET using inkjet printing for the detection of biotin–streptavidin binding events. The OECT was fabricated with polymer-wrapped mono-chiral single-walled carbon nanotubes (SWCNTs), and its water-gate structure reduced the stabilization time to 1 hour for biosensing.^99^

While inkjet printers operate by ejecting droplets of ink in either continuous or discontinuous patterns, each droplet ejected is subject to the coffee ring effect. This phenomenon occurs when the evaporation rates are different between multiple solvent components. In low-viscosity ink, the outer edge of the droplet evaporates faster than its center, resulting in material aggregation at the edges. Various strategies have been proposed to mitigate the coffee ring effect, including solvent concentration adjustments and tailored pattern printing. Hu *et al.* investigated solvent composition to optimize MoS_2_-based inkjet printing and found that using IPA : 2-butanol in a 9 : 1 ratio significantly reduced the coffee ring effect compared with using pure IPA or pure 2-butanol alone (Fig. 3f).^92^ However, the 9 : 1 IPA to 2-butanol solvent was insufficient to fully eliminate the coffee ring effect. Building upon these findings, Sui *et al.* explored the use of a parallel-patterned design to overcome the coffee ring effect and produce homogenous continuous layers. They discovered that, for single line printing, the edges of the lines formed a percolating network that was thicker, while the central areas were thinner and less percolating, often constituting single-layer MoS_2_ or no active material (Fig. 3g). By designing appropriate multi-array patterns, it is possible to create a pattern where the edges of single lines overlap with the inner areas of adjacent lines, building a pattern that has thicker percolating MoS_2_ in the central area with a thinner edge.^93^

Another significant challenge associated with inkjet-printing technology lies in ink formulation. Both the viscosity and material size of the ink are critical for successful printing, requiring extensive efforts to develop new inks suitable for fabrication. The process of adjusting the solvent composition demands considerable experimentation, which can be highly time-consuming. The ink rheology is conventionally evaluated by the inverse Ohnesorge number $Z=\frac{\sqrt{\gamma \rho \alpha }}{\eta }$ which is dependent on the ink’s viscosity (η), surface tension (γ), and density (ρ), and the printer cartridge nozzle diameter (α). Printable inks generally have a Z value ranging between 1–14.^100^ While inkjet printing holds great potential for scalable mass production, nozzle clogging remains a substantial challenge, especially when working with larger particles and high-viscosity solvents.^82^

### Aerosol jet printing

**Fig. 4:**
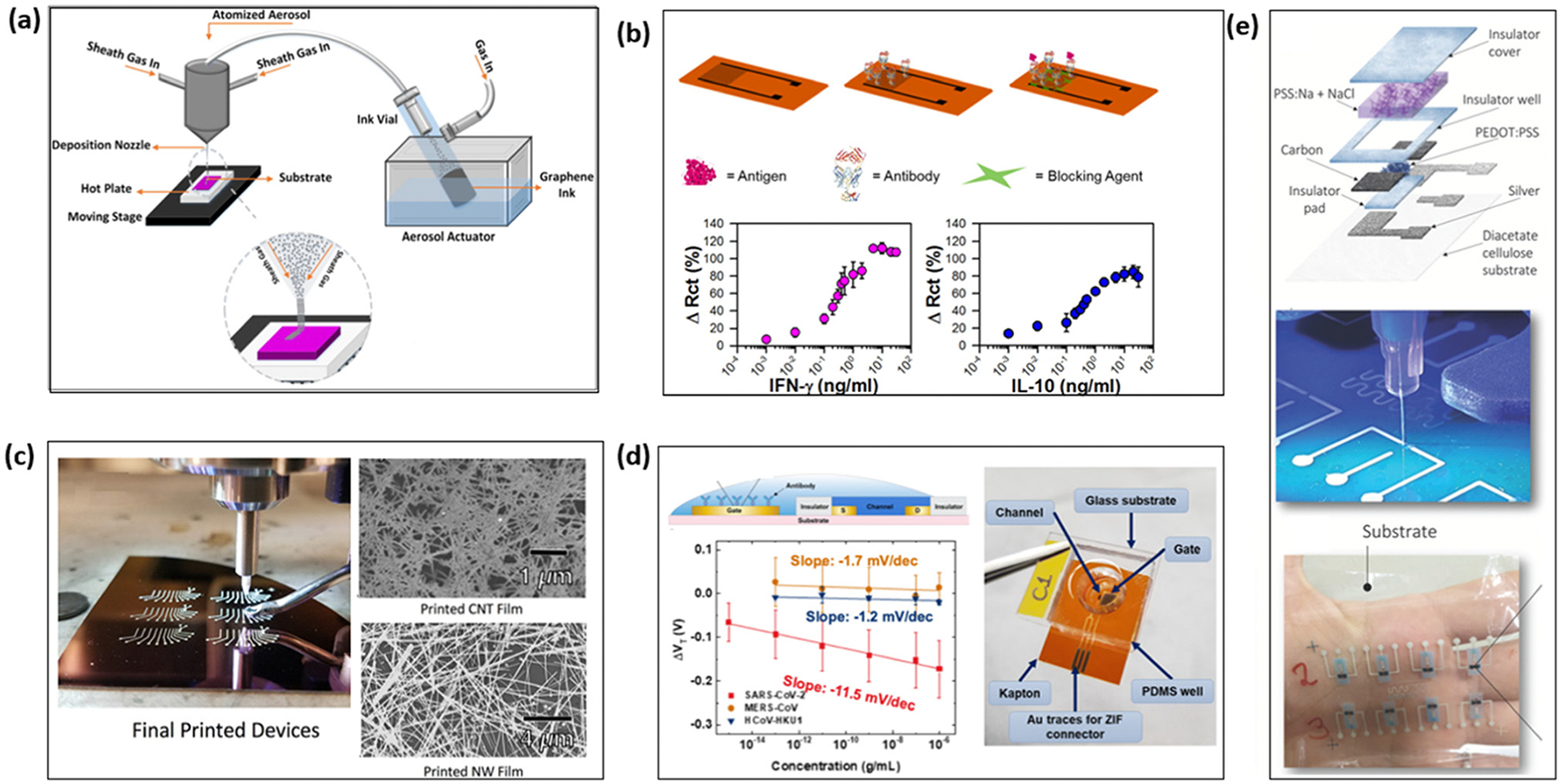
(a) Overview of aerosol jet printing with a schematic delineating the key physical processes involved in aerosol jet printing and an illustration of real printing process.^107^ “Reproduced from ref. 107 with permission from Elsevier, copyright 2015”. (b). Aerosol-jet printing graphene immunosensor for label-free cytokine monitoring in serum.^108^ “Reproduced from ref. 108 with permission from the American Chemical Society, copyright 2020”. (c). Low-temperature aerosol-jet printed carbon nanotube-based transistor.^109^ “Reproduced from ref. 109 with permission from the American Chemical Society, copyright 2018”. (d). Aerosol-jet printed organic electrochemical transistors for point-of-care (POC) SARS-CoV-2 antigen detection.^24^ “Reproduced from ref. 24 with permission from The Royal Society of Chemistry, copyright 2018”. (e). Schematic structure and fabrication of maskless OECTs on biodegradable and compostable substrate.^110^ “Reproduced from ref. 110 with permission from Wiley, Creative Commons Attribution NonCommercial license, copyright 2022”.

Aerosol jet printing (AJP) is another non-contact printing technique developed in recent decades. The AJP process involves aerosolizing ink into micron-scale droplets and delivering them to the substrate through a carrier gas (Fig. 4a). The first stage of aerosol-jet printing is ultrasonic atomization, where the ink is converted into micro-scale size droplets. High-frequency ultrasound (MHz) forms capillary waves on the liquid surface, breaking it into small droplets.^101^ The droplet formation and size distribution is closely related to factors such as crystal frequency, viscosity, surface tension, and ink density.^102,103^ For inviscid liquid, smaller surface tension, higher crystal frequency, and greater ink density typically result in smaller droplet sizes, as described in the droplet formation equation by Boucher and Kreuter.^103^ On the other hand, viscous liquid droplet size depends primarily on viscosity and frequency.^102^ These small droplets undergo rapid evaporation during the delivery process, leading to reduced droplet size. As the carrier gas becomes saturated with the evaporated solvent, the small droplets stabilize during transit. Controlling the composition of low-volatility solvents can help prevent the complete drying of droplets, though this adds significant complexity to the ink optimization process.^104,105^ The second stage involves the carrier gas transporting the micro-scale size droplets from the ink cartridge through a tube to the nozzle. During transit, droplets that collide with the tube walls are lost, and droplet size plays an important role in this process. Large droplets are more susceptible to gravitational settling, while small droplet sizes are more prone to diffusion-induced impingement on the tube walls. The impinged material can build up over time, affecting the deposition rate, causing contamination if multiple materials are used for printing, and potentially clogging.^106^

At the nozzle head, a second gas, known as the sheath gas, is used to surround the carrier gas and help collimate the aerosol stream through the focused nozzle head. This sheath gas also plays a crucial role in preventing material accumulation at the nozzle, ensuring smoother printing operations. The focusing of the aerosol stream is governed by a parabolic velocity profile typical of fully developed laminar pipe flow, as described by the following eqn (3).^106,111^

(3) $$\frac{D_{a}}{D_{n}}=\sqrt{1-\sqrt{\frac{FR}{1\;+\;FR}}}\;\text{where}\;FR=\frac{f_{s}}{f_{a}}$$

Here $f_{a}$ and $f_{s}$ are the carrier (aerosol) and sheath flow rates, $D_{a}$ is the aerosol stream diameter, $D_{n}$ is the nozzle diameter, and FR denotes the focusing ratio. Once the droplets exit the nozzle heads, their inertia causes them to deviate from the gas flow streamlines, resulting in a shift towards the flow center-line. Only droplets that reach the critical size will impact the substrate, forming a broadened deposition pattern. Among these impacted droplets, smaller ones tend to spread over a wider area, creating a more dispersed distribution. The focusing efficiency of this process is a function of droplet size, nozzle diameter, and flow rates.^112,113^

As discussed in the previous sections, several factors critically influence the final resolution of the predesigned patterns in aerosol jet printing. These factors include atomizer power, nozzle speed, ink and substrate temperatures, gas flow rates, and nozzle size. Specifically, the atomizer power and ink properties—such as temperature, viscosity, and composition—predominantly determine both the droplet size distribution and aerosol density. The nozzle speed, also referred to as printing speed, dictates the deposition density of materials, directly influencing both the thickness and the width of line patterns. Additionally, substrate temperature plays a pivotal role in the drying process of inks, which can impact film uniformity, morphology, and overall resolution.^106^ Given the fundamental principles of aerosol jet printing, a variety of inks can be formulated from conductive, dielectric, polymeric, magnetic, or other material types.^114–117^ Conventional ink formulations typically comprise functional materials, binders, and optimized cosolvents with varying volatilities.^118^

One significant difference between aerosol jet printing and inkjet printing is the droplet size. Aerosol jet printers can print out aerosols at micron scales, while inkjet printers typically generate ink droplets from 20–30 μm in size.^84,119^ The smaller aerosol droplet size allows aerosol jet printing to achieve extremely high-resolution printing, with a resolution as fine as 10 μm.^120^ Similar to inkjet printing, aerosol jet printing can print materials onto various substrates, though this often comes at the expense of printing speed and limited control over certain morphologies. Aerosol jet printing has been applied in the fabrication of paper-based thin film transistors (TFTs) at room temperature^121^ and multi-length-scale graphene-coated 3D electrodes.^122^

Given aerosol-jet printing’s high resolution and adaptability, it has been widely utilized for fabricating various nanomaterial-based FET sensors for biomolecular detection over the last few decades. Parate *et al.* fabricated a graphene-based immunosensor for the detection of cytokines, such as interferon gamma and interleukin 10, using aerosol jet printing (Fig. 4b) and achieved detection ranges of 0.1–5 ng mL^−1^ and 0.1–2 ng mL^−1^ in serum, respectively.^108^ The detection of cytokine in serum plays a crucial role in monitoring inflammation in patients and some autoimmune diseases.^123^ As many in-organic inks require additional thermal or chemical post-processing, which is time-consuming and high-cost, studies have been done on the use of aerosol-jet printing to fabricate low-temperature (below 80 °C) stretchable carbon nanotube transistors (Fig. 4c).^109^ The resulting transistor demonstrated an effective mobility of 12 cm^2^ (V s)^−1^ and an on/off ratio exceeding 10^5^. However, the application of aerosol-jet printing in TMD-based FET biosensors, such as MoS_2_-based FETs, remains an area of active research.

Aerosol-jet printing has also been widely combined with conductive polymers for fabricating OECT-based biosensors. Compared with screen-printed OECTs, aerosol-jet printing enables a reduction in channel width to approximately 15 μm and an estimated decrease in the active channel volume by a factor of 40. The all aerosol-jet printed OECT demonstrated decreased overall capacitance and enhanced response speed.^124^ One study demonstrated the application of aerosol-jet printing in POC SAR-CoV-2 detections. In the work, gold (Au) ink was printed as a contact electrode, PEDOT:PSS was printed as a channel material, and PDMS was used as an insulator (Fig. 4d). The device achieved a detection range of 1 fg mL^−1^ to 1 μg mL^−1^ SARS-CoV-2 antigens in PBS and the clinical study reported an overall accuracy of 70% in detecting SARS-CoV-2 antigens from unprocessed patient nasopharyngeal swabs.^24^ Furthermore, aerosol-jet printed OECTs biosensors have also been used for glucose detection.^125^ In this application, glucose oxidase is used for probe functionalization. The enzymatic reaction of glucose oxidase and glucose generates oxygen, H^+^ and e^−^. The resulting cations can penetrate the channel, compensating the negative charges in PEDOT:PSS and resulting in a reduction in drain current. Granelli *et al.* demonstrated maskless all-printed OECTs on biodegradable and compostable substrates, offering a cost-effective fabrication alternative to traditional multi-step photo-lithographic microfabrication.^110^ The schematic structures and fabrication process are shown in Fig. 4e. The fabricated sensor can be used for ion detection and real-time monitoring with a sensitivity up to 506 mV dec^−1^.

Aerosol-jet printing technology holds significant potential for directly fabricating probe-immobilized electrodes with biological and metal mixtures. Biological molecules such as proteins, enzymes, and high molecular weight DNA have demonstrated the ability to retain their activity after printing, making this technique promising for biosensor applications.^126^ Currently, most biosensors require an additional immobilization step to anchor the functional probes before the sensing process. If the immobilization step could be integrated into the fabrication process, then it can significantly reduce the time required for detection in fully printed biosensors. The development of such a technique may ultimately allow for on-site printing of customized biosensors tailored to specific detection needs in the future.

Aerosol-jet printing shows great promise in the fabrication of biosensors; however, it faces several challenges that hinder its widespread adoption. Although a wide range of inks can be employed for printing, the optimization of inks is often a sensitive and time-consuming process which requires extensive empirical trials.^106,127^ Even with high quality inks, establishing and maintaining the predesigned ink stream remains a significant challenge in aerosol-jet printing, primarily due to the absence of a real-time monitoring and feedback system.^118^ Moreover, for industrial-level production, where reproducibility is paramount, aerosol-jet printing must improve its throughput.^69^ While aerosol-jet printing offers a higher printing resolution and a thinner materials deposition compared with inkjet printing, screen printing and even digital light processing, its deposition speed is several times slower than these alternative methods.

### Digital light processing

**Fig. 5:**
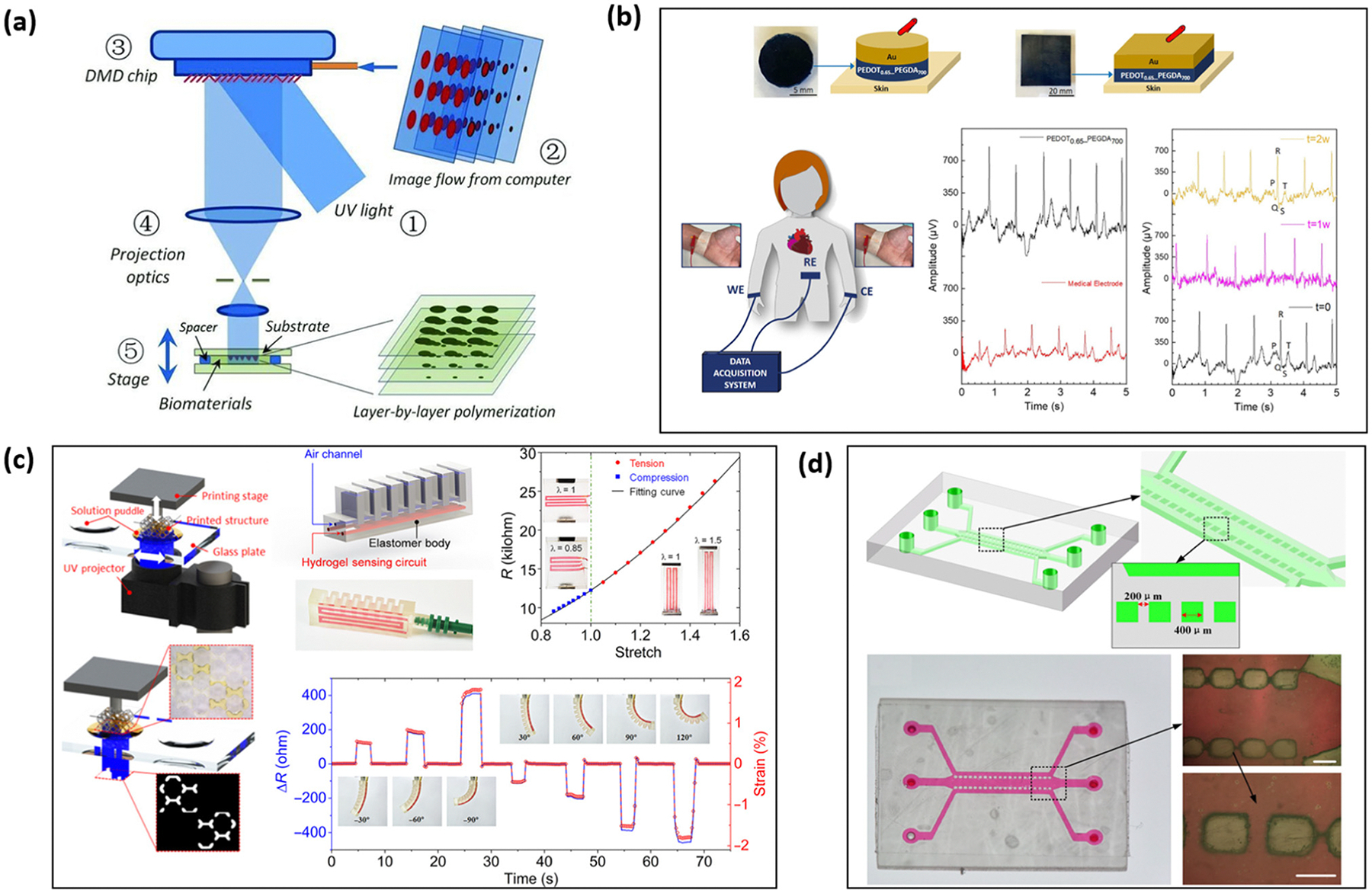
(a) Schematic of the digital light processing mechanism. The digital micro-mirror array device controls the projection light to polymerize materials layer-by-layer to obtain a high-resolution predesigned structure.^129^ “Reproduced from ref. 129 with permission from Wiley, copyright 2012”. (b). Digital light processing PEDOT-based sensor for human electrocardiography (ECG) and electromyography (EMG) detection.^130^ “Reproduced from ref. 130 with permission from the American Chemical Society, CC-BY-NC-ND 4.0, copyright 2021”. (c). Printed soft pneumatic actuator with a hydrogel strain sensor.^133^ “Reproduced from ref. 133 with permission from Science Advances, Creative Commons Attribution NonCommercial License 4.0 (CC BY-NC), copyright 2021”. (d). Fabrication of microfluidic chips with a DLP-based printer.^134^ “Reproduced from ref. 134 with permission from MDPI, Creative Commons Attribution (CC BY) license, copyright 2023”.

Digital light processing (DLP) is an emerging photo-polymerization technique that resembles the stereolithography technology patented by Chuck Hull. This method employs the projection of light to polymerize materials layer-by-layer to obtain a high-resolution predesigned structure.^63,128^ The core hardware of DLP printers is a digital micro-mirror array device (DMD), which consists of a programmable micro-opto-electrochemical chip with an array of micro-mirrors (Fig. 5a). The *X*–*Y* resolution of the printed structure is defined by the projection from the micro-mirror chips and can achieve a precision down to 3–5 μm. The *Z*-axis resolution, however, is limited by the material refill process.^51^ Chen *et al.* has developed a DMD that can continuously move along the *Z*-axis during printing to enable smooth side-wall printing.^129^ DLP has been widely used in the bioengineering field to produce artificial tissues, medical devices, and targeted drug delivery systems. The recent advances in conductive photocurable polymers have paved the way for using DLP to produce high-resolution, stretchable biosensors. Traditional fabrication techniques, such as line-to-line and point-to-point inkjet printing, aerosol-jet printing, or screen printing of photocurable conductive polymers, often require an additional post-photo-polymerization step after printing to finalize the patterned structure.^130,131^ This process is typically tedious and time-consuming. In contrast, DLP enables one-step photo-polymerization of an entire 2D plane pattern, simultaneously printing and curing the pattern. This integration offers significant advantages in terms of printing resolution, quality control, efficiency, and operational conditions.^132^

Nowadays, the conventional fabrication of transistor-based sensors, such as FETs and OECTs, still relies on a layer-by-layer process where the transducing, electrode, and dielectric layers are produced separately, which is time-consuming. However, the 3D DLP technology offers the potential to revolutionize this approach by enabling the creation of a complete biosensor—comprising transducing, conductive, and dielectric layers—in a single step. This makes DLP a promising candidate for the high-throughput fabrication of complex 3D biosensors using conductive polymers. Despite the potential of DLP technology, the direct combination of DLP and transistor-based biosensors is still very limited. One specific example of DLP-printed bioelectronics is human electrocardiography (ECG) and electromyography (EMG) recording sensors (Fig. 5b). These sensors are fabricated using PEDOT:PSS dispersed within a matrix of poly(ethylene glycol) diacrylate (PEGDA), ethylene glycol (EG), and a photoinitiator. The printed bioelectrodes exhibited potential for long-term (2-week) ECG and EMG recordings, with signal quality comparable to that of commercial Ag/AgCl medical electrodes.^130^ Another example is the fabrication of flexible strain sensors, which are essential components in soft robotics and artificial muscle systems.^135^ Xiao *et al.* fabricated a flexible sensor with hybrid DLP-3D printing, where MWCNTs served as a sensing unit, and selective electrostatic self-assembled silver nanowires were selected as interconnectors. The woodpile structure design enhanced the sensitivity by approximately 9 times compared with the solid counterpart and the fabricated 4 by 4 sensor array demonstrated the ability to detect external pressure distribution.^131^ Additionally, Ge *et al.* utilized the DLP technology to fabricate complex 3D conductive hydrogel-integrated devices and systems for strain detection. A 3D soft pneumatic actuator (SPA) strain detector (Fig. 5c) was printed in one step using a high-water-content and highly stretchable PEGDA-based hydrogel.^133^ As the strain detector bent, changes in ion conductivity within the hydrogel were measured *via* resistance variations.

Because of the compatibility with conductive polymers, DLP has also been used in fabrication of functional device integration, such as microfluidic channels for biosensors. Qiu *et al.* tested out the fabrication of microfluidic channels through the DLP process (Fig. 5d), achieving a 10% error for 300 μm microfluidic channels and less than 5% error for microfluidic channels larger than 400 μm.^134^ In the coming decades, as health monitoring receives increasing attention, DLP has enormous potential for developing integrated polymer-based sensor and microfluidic platforms for both sample collection and analysis. Early examples of such wearable body health monitoring devices include wearable sweat analyzers and glucose detectors.^136,137^ Wu *et al.* utilized DLP for the fabrication of epidermal microfluidic devices capable of collecting and analyzing sweat.^137^ The DLP technology enables the fabrication of a true 3D design space for microfluidic devices, with previously inaccessible complex structures. In a field study, up to 50.8 μL of sweat was reported to be collected within a 40 minute biking exercise.

Despite these advancements, the application of DLP technology in diagnostic biosensors remains limited compared with its broader use in fields such as artificial organ development. A key limitation is that most DLP inks available are polymer-based, restricting material options for biosensor fabrication. Thus, expanding the range of compatible ink materials is essential for DLP to become competitive in this field. Other challenges include optimizing ink compositions, minimizing material waste, and improving printing resolution to enhance the performance and scalability of DLP-fabricated biosensors.

In conclusion, additive manufacturing techniques significantly accelerate the biosensor development given their fast and economical prototyping ability, along with their versatilities in material selection and surface printing. Each printing technique possesses unique advantages and is suitable for specific biosensor fabrication. Table 1 summarizes the materials, benefits and limitations of four printing techniques, along with spin coating, chemical vapor deposition (CVD) and conventional lithography for biosensor fabrication. Of the seven fabrication techniques, conventional lithography is considered the state-of-the-art technology for FET fabrication and is already fully industrialized. While conventional lithography enables the production of high-performance FET sensors with excellent uniformity, it requires complex procedures and clean-room environments, and is restricted to silicon wafers and silicon-based semiconductors, making it less suitable for developing novel transistor-based biosensors. Spin coating and CVD are two other widely used methods for fabricating transistor semiconducting channels, offering a broader range of material options. However, neither of these methods can independently fabricate the entire FET. In contrast, printing-based fabrication methods offer a balanced trade-off between cost and quality, ease of prototyping, reduced fabrication time and less instrument reliance, and single-step fabrication capability along with versatility in material and substrate selection. Among the four additive manufacturing techniques presented here, the DLP printing technology is primarily limited to photosensitive polymers, representing an emerging technique, while the other three more mature printing technologies are compatible with a wider range of materials. Of the three mature printing methods, screen printing is the most cost-effective, whereas aerosol-jet printing is the most time-consuming. In terms of quality, aerosol-jet printing achieves the highest resolution of the three, with screen printing being the least precise. The biosensors developed through these methods can integrate additional functionalities to meet the growing demands in public health such as onsite analysis, real-time detection, and large-scale pathogen monitoring.

## 3. Transistor-based biosensor device integration for real-life application

With the advances of clinical diagnosis, modern biosensing is gradually shifting from off-site laboratory tests to on-site POC real-time diagnosis. Various studies have focused on reducing the cost and improving the performance of transistor-based biosensors, which are the ultimate goals of traditional off-site laboratory tests. However, on-site real-life biosensing applications not only require high performance of the sensing platforms but also have to meet diverse needs in real-life applications through the integration of various functional units. Considering the WHO criteria for POC tests: affordable, sensitive, specific, user-friendly, rapid and robust, equipment-free, and deliverable to end users, the ultimate goal for translating biosensors from laboratory to commercial usage should include miniaturization into a handheld device, simplified processing and testing procedures, and integration of samplers and the IoT. This section will discuss common device integrations required for transistor-based biosensors to meet needs in real-life biosensing.

### Integration into lab-on-a-chip for portable devices

There has long been significant interest in integrating all analytical components into a miniaturized device for real-time biomolecule monitoring in practical applications. From a public health perspective, there is a growing concern about food and water safety, and miniaturized devices capable of detecting harmful organisms in everyday life would be highly valued. From a healthcare perspective, patients seek to monitor specific biomolecule levels to maintain and promote their health. For example, for patients with diabetes, a common disease associated with substantial morbidity worldwide, continuous glucose monitoring (CGM) is essential for maintaining glycemic control and reducing the incidence and duration of hypoglycemia for both type I and type II diabetes.^138^ To meet these needs, a wide variety of portable glucose biosensors have been developed, and the transcutaneous CGM market has substantially expanded in recent decades.^139^

**Fig. 6:**
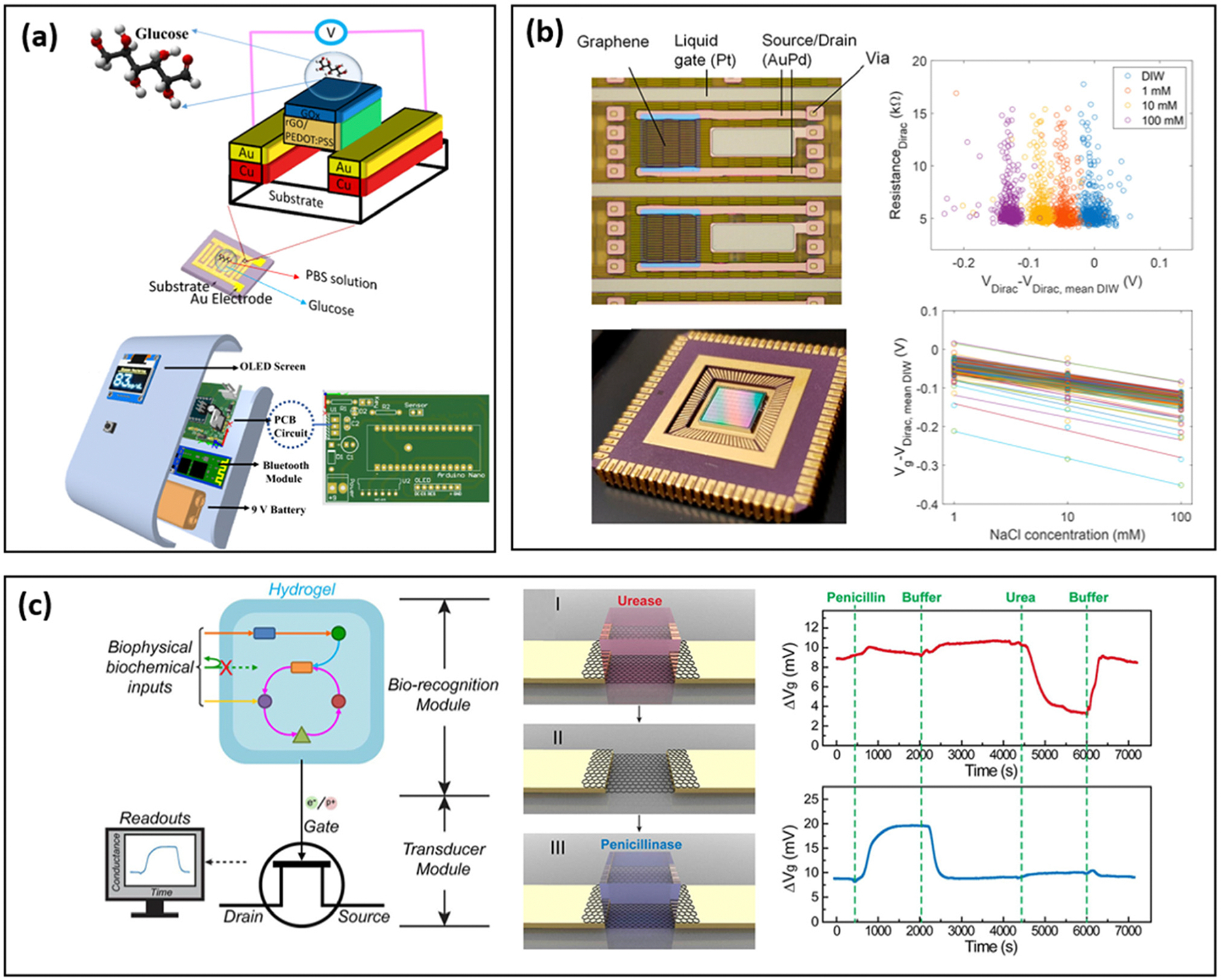
(a). FET-based portable glucometer on interdigitated electrodes and printed circuit board.^141^ “Reproduced from ref. 141 with permission from Springer Nature, Creative Commons Attribution (CC BY) license, copyright 2023”. (b). Wafer-scale graphene FET biosensor arrays on CMOS for sodium chloride detection.^143^ “Reproduced from ref. 143 with permission from the American Chemical Society, CC-BY 4.0, copyright 2023”. (c). Modularized FET with a separated design of biorecognition units and transducer units.^144^ “Reproduced from ref. 144 with permission from the American Chemical Society, copyright 2019”.

Given the demands of portable miniaturized devices, significant research has focused on translating laboratory-based transistor biosensors into compact and portable market-ready devices.^139,140^ Lab-on-a-chip is the technology that facilitates the miniaturization of transistor-based biosensors into a portable chip with the embedment of various sensor components. Monfared Dehbali *et al.* developed an FET-based portable glucometer using heterostructure materials on interdigitated electrodes.^141^ The device integrates electronic modules, including a power supply, analog-to-digital converter, OLED screen, and wireless transmitter, into a single printed circuit board, which is then packaged into a portable glucometer (Fig. 6a). This glucometer operates based on a glucose-oxidase enzyme reaction, wherein electrons produced during the enzymatic process recombine with hole carriers in a reduced graphene oxide (rGO)/PEDOT:PSS composite, leading to a decrease in channel conductivity. The developed sensor showed a limit of detection of 1 μM and exhibited a high clinical accuracy compared with commercial devices. In a related study, Chen *et al.* successfully demonstrated a portable, rGO FET-based device platform for rapid water quality detection.^142^ The sensor platform constitutes of a miniaturized microcontroller for pulse generation, signal measurement and recording, and an rGO-based sensor chip. The sensor achieved a limit of detection down to 2.5 ppb lead in DI water, which is significantly below the US Environmental Protection Agency (EPA) maximum contaminant level (MCL) of 15 ppb for drinking water.

For laboratory-based biosensors to transition into real-world applications, the ability to support mass-production is a critical criterion. Standardized fabrication processes can significantly reduce the per-device cost, minimize device-to-device variability, and increase the device yield in mass production. Two of the major challenges hindering the transition of laboratory-developed biosensors into practical applications are low device yield and significant batch-to-batch variability. Extensive research has been conducted to address these issues and optimize the device performance. One strategy to improve the yield and reduce the batch-to-batch variability is through proper sensor structure design and precise control over fabrication parameters. Soikkeli *et al.* demonstrated a wafer-scale graphene-based FET design on a complementary metal–oxide semiconductor (CMOS) substrate (Fig. 6b) and achieved more than 99% yield with less than 30% device-to-device variation for sodium chloride sensing, indicating the potential for mass-production of this specific lab-on-a-chip design.^143^ The biosensors exhibited a response to sodium chloride ranging from 1 mM to 100 mM with a 42 mV dec^−1^ sensitivity. Similarly, Liang *et al.* showcased a wafer-scale carbon nanotube-based FET with an yttrium oxide (Y_2_O_3_) insulation layer on a silicon/silicon oxide substrate, reporting 100% fabrication yield and outstanding semiconducting properties.^145^

During the optimization of transistor-based biosensors, research has revealed that the simultaneous usage of nanomaterials as both the transducer and sensing membrane can lead to signal complexation, potentially reducing the sensitivity and selectivity. When analytes bind to probes, they may interact ambiguously with the transducer material, leading to effects such as local scattering and chemical gating, which can drive the sensor signal toward opposite directions. A potential solution to this issue is to incorporate insulating layers to decouple these ambiguous interactions to a univocal interaction. Liang *et al.* demonstrated that while adding an Y_2_O_3_ dielectric layer reduces the semiconducting performance of the FET, such as on-current magnitude and transconductance, it enhances the overall sensor detection capability, achieving an LOD of 60 aM for DNA and 6 particles per mL for microvesicles.^145^

To further improve the reusability and cost-effectiveness of FET-based sensors, separating the biorecognition modules from the transducer module could be a promising approach. This modular separation allows for the biorecognition components to be replaced independently, thereby reducing overall test costs while facilitating the industrial application of biosensors. Dai *et al.* reported a hydrogel-based replaceable biorecognition module that can be assembled and disassembled onto the FET-based biosensors.^144^ This sensor can detect penicillin at a concentration as low as 0.25 mM. The hydrogel-based replaceable biorecognition module can be mass-fabricated through 3D printing in a cost-effective manner while offering high customizability (Fig. 6c). Jang *et al.* developed a commercialized silicon FET based floating gate platform for label-free SARS-CoV-2 detection using an rGO-based sensing membrane.^146^ The rGO-based refloating gate membrane provides the anchor site for probes and can be easily fabricated through techniques such as drop-casting and inkjet printing. Because the impedance of the metal–oxide-semiconductor field-effect transistor (MOSFET) is at least 100 times higher than that of the remote floating gate structure, the thickness of the rGO sensing interface has a negligible effect on the sensing signal, which provides the basis for high reproducibility of this system. The fabricated sensor demonstrates rapid detection for SARS-CoV-2 in nasal swab samples with 90.6% accuracy and an LOD that is 10 000 fold-lower than enzyme-linked immunosorbent assays.^147^

Besides improving the device yield and reducing the device-to-device variation, researchers have increasingly focused on the selection of substrates for mass production. Traditional substrates used in the laboratory manufacturing, including silicon, paper, glass, and polydimethylsiloxane (PDMS), are not ideal for large-scale production. Although silicon substrates benefit from their well-established industrial infrastructure, they are prohibitively expensive for producing cm-scale devices. Paper substrates have emerged as novel materials in this field, given their cost-effectiveness, portability and ease of use; however, further research is required to fully unlock the potential of paper as viable substrates. PDMS, while biocompatible, cheap, and transparent, also lacks the capacity for electronic integration, similar to glass substrates.

Printed circuit boards (PCBs) have been explored as a promising alternative substrate for lab-on-a-chip manufacturing due to their well-established industry system and compatibility with both electronic integration and microfluidic embedding. The incorporation of microfluidic channels onto the PCB can be achieved using standard industry equipment and computer-aided design (CAD) tools. Furthermore, the manufacturing constraints for microfluidic channels on PCBs align well with the typical size requirements of microfluidic channels (50 μm–100 mm).^148^ Papamatthaiou *et al.* fabricated BioFET for DNA quantifications with printable graphene ink on a lab-on-PCB system and achieved a limit of detection of 1 nM for target complementary DNA.^149^ The entire sensor was fabricated by drop-casting graphene inks onto preprinted PCB structures, eliminating the tedious lithography steps typically required for FET fabrication. The lab-on-PCB platform has demonstrated its feasibility for seamless mass production when integrated with additive manufacturing technology.

The final step for a portable biosensor to function effectively is the bioreceptor modification of the sensing surface. Transistor-based biosensors, such as FETs and OECTs, require the immobilization of sensitive and selective probes on the sensing surface to capture target analytes and generate a recognizable signal. Commonly used probes in bioelectronic applications include antibodies, antibody fragments, and DNA/RNA-based probes. Traditional antibodies, which are immuno-globulin proteins approximately 150 kDa in size, offer high affinity toward target proteins and have been widely used for detecting biomolecules such as cells, bacteria, viruses, cancer markers, and proteins for over 70 years. Even with the advent of various newly developed bioprobes, antibodies are still regarded as the “gold standard”. Examples of antibody-based biosensors include, but are not limited to, COVID-19 spike protein sensors, exosomes sensors, and Ebola sensors.^44,150,151^

Although antibodies are the most frequently selected probes, concerns such as batch-to-batch variation remain to be addressed. Recently, there has been considerable interest in using antibody fragments as a potential replacement for traditional antibody probes. Antibody fragments not only retain the specificity and high binding affinity of antibodies, but are also smaller in size and amenable to various modifications that can stabilize production and minimize batch-to-batch variation.^152–154^ However, the production of antibody fragments is still predominantly lab-based, with only a limited choice of expensive commercial options, rendering them less ideal probe choices for the practical application of POC biosensors.

Aptamers, which are single-stranded DNA or RNA oligomers, can specifically bind to cognate biomolecules through their unique tertiary structure.^155^ The high specificity, binding affinity, simplicity of synthesis, and diverse structural characteristics make aptamers promising candidates for biosensor probes and a potential replacement for antibodies in the near future.^156^ Additionally, some biosensors directly utilize DNA/RNA as probes to detect the presence of specific DNA/RNA sequences.

### Integration with microfluidic channels

To address the need for simplified processing and a higher accuracy, biosensors can be integrated with microfluidic channels which enable detection in a continuous fluid with controlled flow rates. Microfluidics, which is also known as micro-total analysis systems, refers to integrated platforms that combine preparation, reaction, separation and detection within a single chip.^157^ Microfluidic platforms and lab-on-a-chip designs build the cornerstone for the further translation of transistor biosensors. Microfluidic chips constitute engraved microchannels, typically ranging from 10–100 μm in size, and are fabricated using various substrates such as silicon, glass, and polymers like polydimethylsiloxane (PDMS) and polymethyl methacrylate (PMMA). A key function of microfluidic systems is to realize precise fluid manipulation and analysis while minimizing sample consumption.^158^

**Fig. 7:**
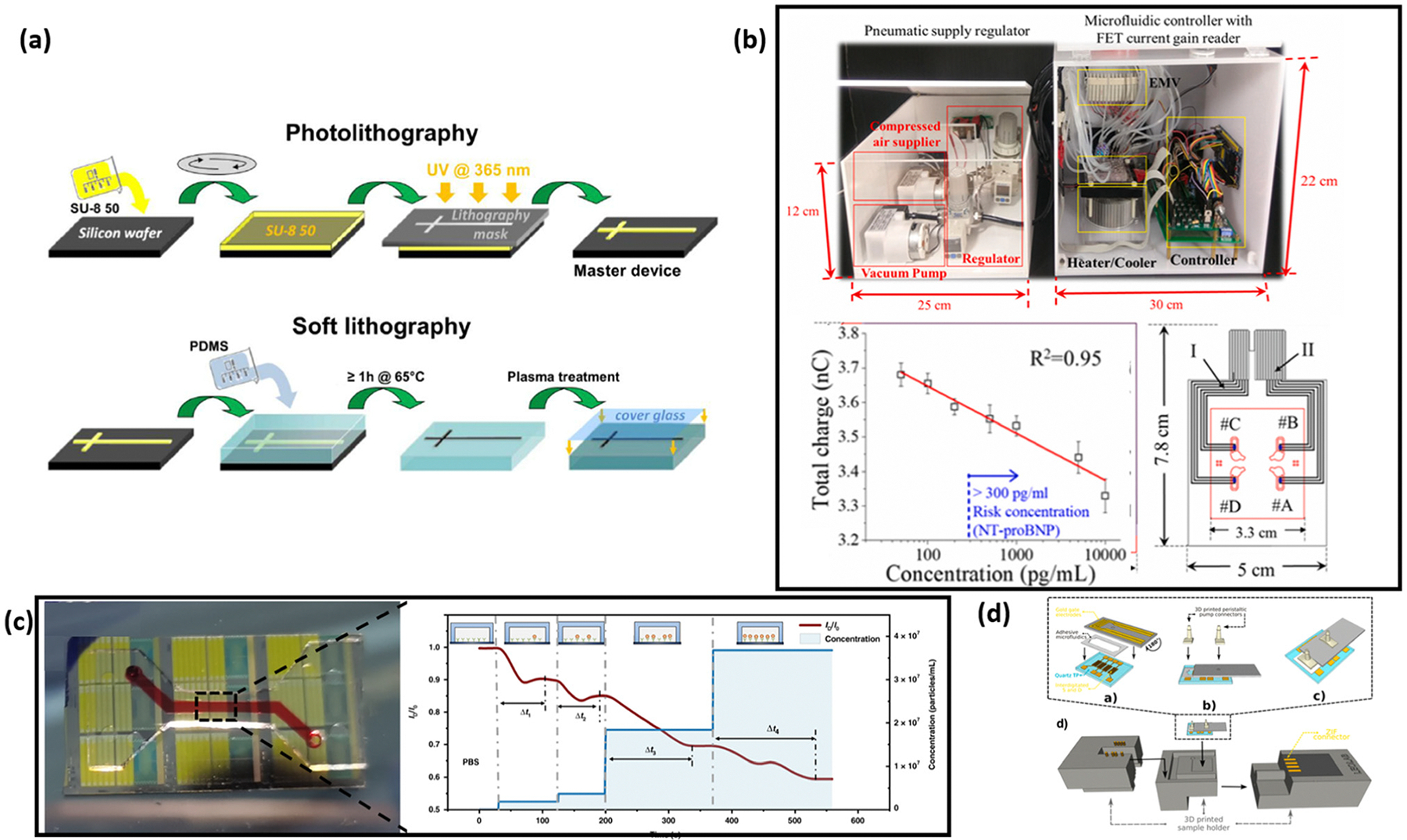
(a). Schematic of soft lithography-based fabrication of microfluidic channels.^163^ “Reproduced from ref. 163 with permission from The Royal Society of Chemistry, copyright 2013”. (b) Integrated FET sensor with microfluidic channels for automatic sampling and simultaneous detection of biomarkers for cardiovascular diseases.^164^ “Reproduced from ref. 164 with permission from Elsevier, copyright 2019”. (c) Microfluidic channel integration with silicon nanowire bio-FET for electrical and label-free detection of cancer cell-derived exosomes.^46^ “Reproduced from ref. 46 with permission from Springer Nature, Creative Commons Attribution (CC BY) license, copyright 2022”. (d). Microfluidic channel embedment for multi-gate detection of TNFα.^165^ “Reproduced from ref. 165 with permission from the American Chemical Society, CC-BY 4.0, copyright 2020”.

PDMS has been widely used in the fabrication of microfluidic channels for bio-related research, such as cell-screening and biochemical assays, due to its affordability, ease of molding, gas permeability, biocompatibility and low autofluorescence.^159^ Depending on the material properties, the fabrication methods of microfluidic channels vary. The fabrication methods can be classified into two categories: material removal techniques or material deposition techniques. These techniques include chemical, mechanical, laser-based, and printing processes.^159^ For chemical processing, the most commonly adopted techniques are wet and dry etching, along with electrochemical discharge machining, which are used for manufacturing microfluidic channels in both glass and silicon substrates.^160,161^ In mechanical processing, soft lithography is one of the most popular methods for fabricating microfluidic channels for biosensor applications.^162^ Soft lithography typically involves creating a hard mold, pouring liquid polymer into the mold, heat-curing, and peeling off the final product (Fig. 7a). For laser-based processes, stereolithography and two-photon polymerization are well-known for producing high-resolution patterned products. Lastly, 3D printing, which includes inkjet-printing, fused deposition modeling, and multi-jet modeling, has recently gained significant attention and success in the fabrication of microfluidic channels.^159^

Microfluidic platforms have been widely applied in biomedical engineering,^166^ chemistry,^167,168^ medicine,^169,170^ biology,^171^ and environmental science.^172^ The integration of a microfluidic system is a critical step in translating biosensors from laboratory research to real-world applications. Microfluidic technology allows for the incorporation of multiple functional modules into a compact device, enabling complex and precise fluid manipulations that achieve high sensitivities. The advantages of incorporating microfluidic platforms into biosensor systems include, but are not limited to: (1) enabling the detection of analytes in continuous flow and the simultaneous detection of multiple analytes; (2) streamlining complex assay protocols, such as mixing and washing; (3) minimizing sample volumes and reducing detection times, which further lowers costs when using expensive reagents; (4) providing a foundation for automated, high-throughput measurements and further integration of functions like sampling; (5) potentially enhancing the sensitivity by increasing the surface-to-volume ratio; and (6) miniaturizing the detection system for portability, disposability, and real-time detection.^158,164,173–176^ The high controllability and versatility offered by microfluidic channels make them a corner-stone for the development of next-generation POC biosensors.

Biosensors integrated with microfluidic channels can achieve automatic sample mixing and washing with minimum human operation. Sinha *et al.* demonstrated the design of a fully automated detection system based on FET biosensors integrated with microfluidic platforms for the detection of multiple cardiovascular biomarkers (Fig. 7b).^164^ In this system, all microfluidic control processes—including sample mixing and washing away excess materials—are programmed to ensure full automation. Since aptamers and ssDNA are used in the detection, both a heater and a cooler are required, which results in a size increase of the overall system. The entire automated system can be embedded in boxes measuring 25 cm × 12 cm × 15 cm and 55 cm × 25 cm × 22 cm. This research high-lights the potential of microfluidic channel-integrated FET-based biosensor systems for fully automated detection. Zhao *et al.* also demonstrated that the integration of microfluidic channels allows for the functionalization of the sensing surface to occur simultaneously with the detection of analytes (Fig. 7c).^46^ Many FETs in laboratory settings utilize a drop-casting method to functionalize the sensor surface, which is crucial for enabling analyte binding and detection. However, this drop-casting method requires human intervention, which can hinder the full automation of systems. The introduction of microfluidic-based channels provides the basis for eliminating human intervention, allowing for fully automatic functionalization of the sensing surface and detection in one step. By removing the need for pre-functionalization, the shelf life of the biosensor can be significantly extended, broadening its potential applications.

Microfluidic channels have also been used as chambers for FET-based biosensors, which paves the way for simultaneous monitoring of multiple analytes with sensor arrays. While most organic electrolyte-gated FET reported in the literature are single-gated, Parkula *et al.* integrated OECTs with microfluidic channels and enabled the multi-gate detection of TNFα (Fig. 7d), achieving an LOD as low as 3 pM.^165^ A handheld device holder is designed using 3D printing technology to facilitate the easy installation and replacement of sensor chips, showcasing the potential of transistor-based biosensors for portable on-site detection applications. The ability to simultaneously detect multiple analytes, combined with the potential for full automation, positions the integration of microfluidic channels as a crucial component of biosensing platforms. The integrated biosensing platform can be further combined with advanced systems like samplers, smartphones and even cloud-AI analysis to enhance functionality and versatility. Microfluidic channels have also been effectively used to direct the sample solution to partly cover the channel material, which induces differences in electrical properties between the covered and uncovered areas. Tsang *et al.* demonstrated that using microfluidic channels to partly cover the graphene channels can induce an additional minimum alongside the original Dirac point of the graphene, enabling the sensitive detection of exosomes down to 0.1 μg mL^−1^ in 30 minutes.^151^

Several challenges arise in integrating microfluidic channels with FET-based biosensors, including miniaturization and packaging, material limitations, cost, device-to-device variation, and overcoming complexities in continuous flow detection.^177–179^ Miniaturization and packaging specifically refer to miniaturizing microfluidic platforms to a size that is compatible with biosensor chips and effectively packaging them onto the sensor. Currently, there is no standardized process for packaging microfluidic channels with biosensors. Device-to-device variation is another major concern, as consistency across different devices is difficult to achieve. Additionally, high fabrication costs further hinder the wide-spread adoption and commercialization of these biosensors. Recently, 3D printing has shown promise as a method for standardizing the fabrication of microfluidic systems, offering a potential solution to this problem.^180,181^ Material limitations also present challenges, particularly for application-specific uses. For instance, microfluidic channels designed for wearable devices must be fabricated from biocompatible materials to ensure safety when in contact with the human body.

When applying microfluidic-integrated FET sensors for sensing under physiological environments, such as for biomedically related applications, high-salt solutions can cause a screening effect known as Debye screening, which prevents the detection of analytes beyond the Debye length. The Debye screening length can be down to 1 nm in bio-fluid environments.^182^ For example, when detecting negatively charged molecules like streptavidin or DNA, the analytes become surrounded by positively charged ions from the high-salt solution due to electrostatic interaction. Beyond the Debye length, the number of surrounding positive charges approaches the number of negative charges on the analytes, effectively neutralizing the charge and preventing the FET from sensing any signal.^183^ The Debye length ($\lambda_{D}$) for aqueous solutions at room temperature can be calculated using the following eqn (4):

(4) $$\lambda_{D}=\frac{1}{\sqrt{4\pi l_{B}\sum_{i}\rho_{i}{z_{i}}^{2}}}$$

where $l_{B}$ is the Bjerrum length = 0.7 nm, and $\rho_{i}$ and $z_{i}$ are the charge density and the valence, respectively.^183^ With the microfluidic channels integration, Krivitsky *et al.* proposed an innovative approach to overcome the Debye length problem in whole blood detection by measuring the dissociation between antigens and probes under high-speed flow, rather than focusing on the binding of antigens to probes.^184,185^ By washing with sensing buffer after the binding event, it has been demonstrated that specific antigens will dissociate slower than unspecific antigens under a fast speed flow, resulting in a detectable signal difference. This dissociation-based detection mechanism opens up new possibilities for addressing the challenges posed by the Debye length in biosensing applications.

The integration of microfluidic systems with biosensors enables the detection of analytes in a complex continuous flow. Since most biosensors are designed to detect analytes in a stagnant environment, overcoming the hydrodynamic forces that prevent analytes from binding to the sensing region becomes a major challenge in continuous flow applications, especially when particles are moving at high flow speeds. To address this, electrokinetic techniques such as AC electro-osmosis (EO) and dielectrophoresis (DEP) have been adopted for the separation of single cells within microfluidic channels.^186^ Han *et al.* demonstrated the use of combined AC-EO and DEP techniques to concentrate cells in a continuous flow, effectively overcoming the hydrodynamic forces and enhancing the detection efficiency of FET sensors.^187^ A 14.6-fold increase in fluorescent bead concentration was observed at the detection region using AC-EO and DEP combined techniques. Moreover, the system achieved an LOD of 150 CFU mL^−1^ for S. *aureus* in 0.01× PBS within 35 min, exhibiting high selectivity and specificity against *S. epidermidis*, *E. coli*, and *B. subtilis*.

### Integration with samplers

Pathogen surveillance has become a critical focus, particularly following the outbreak of new epidemics. The recent SARS-CoV-2 pandemic reminds us that we are dramatically underestimating our vulnerability to pathogens and the importance of a rapid and accurate aerosol detection system for pathogenic organisms.^188^ In many countries, pathogen surveil-lance has been untimely and lagged behind the rapid spread of COVID-19. As a result, public health interventions like isolation, contact tracing, and quarantine were ineffectively coordinated to control the spread in the early stages.^189^ Studies investigating virus concentrations in both indoor and outdoor environments have consistently shown that crowded indoor spaces, such as airports and buses, exhibit significantly higher virus concentrations than outdoor settings.^190–192^ This finding underscores the importance of indoor monitoring over outdoor surveillance in open areas, particularly in densely populated environments. For example, Zhang *et al.* have conducted an environmental surveillance suggesting that approximately 100 more COVID-positive cases occurred during weeks with positive environmental samples compared with weeks without positive samples.^190^

During the COVID-19 pandemic, nasal swab sampling followed by a PCR-based sequencing test was considered the gold standard for pathogen detection. However, this PCR-based method relies on benchtop sequencers in a laboratory setting, limiting its applicability for automated pathogen monitoring. Although some portable sequencers have been developed, their high cost hinders the establishment of a widespread automated pathogen monitoring network.^193^ Transistor-based biosensors hold tremendous potential for automated pathogen monitoring networks when integrated with automated samplers, owing to their electrical signal output, miniaturization potential, and suitability for mass production. With proper sampler integration, transistor-based biosensors can offer a highly competitive POC real-time detection method, as compared with the current PCR-based gold standard, which typically requires several hours to days to deliver results.

**Fig. 8:**
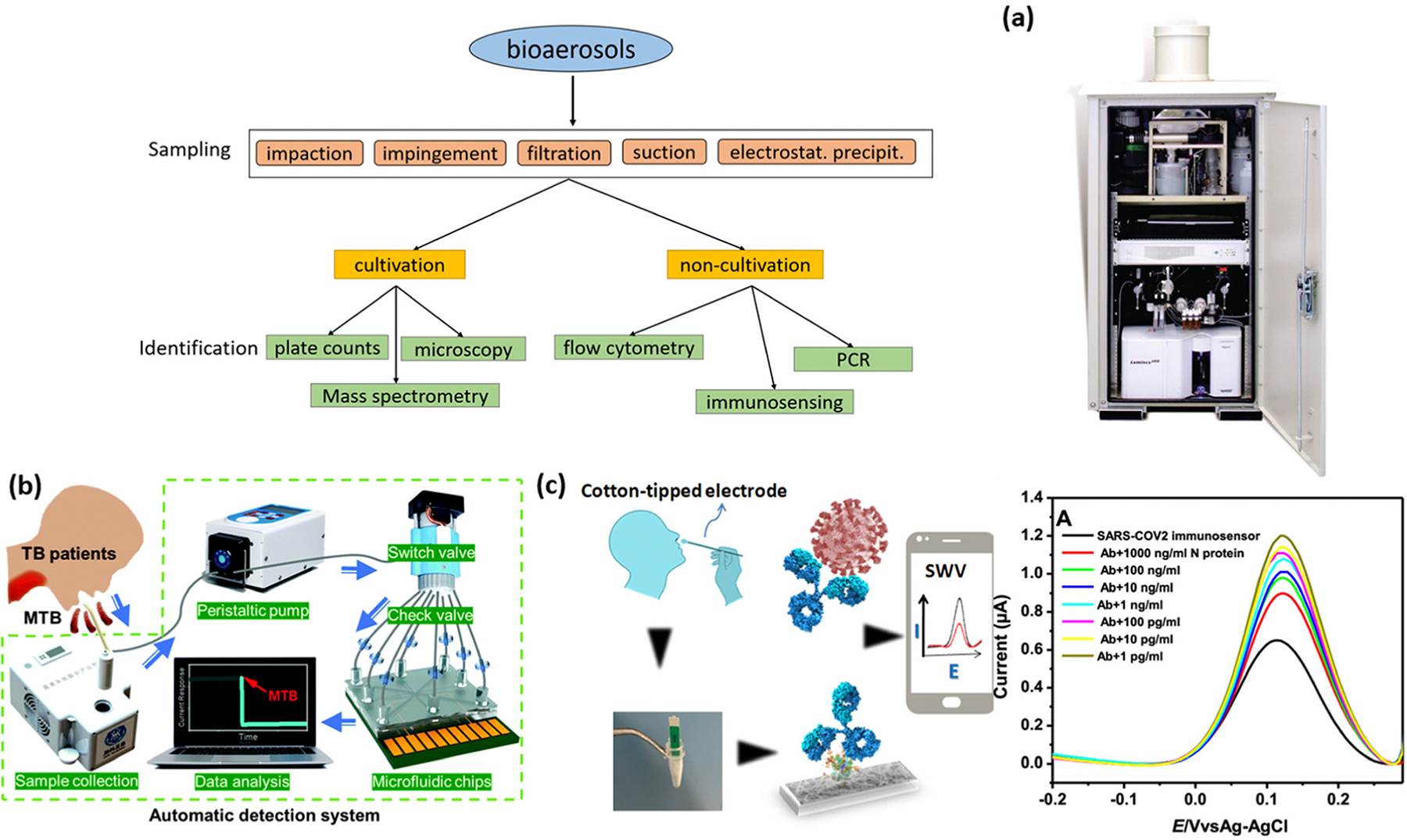
Schematic of common sampling-to-detection methods for bioaerosols. (a). Illustration of the autonomous pathogen detection system (APDS) developed in 2008 for environmental monitoring of biological threat agents.^201^ “Reproduced from ref. 201 with permission from the American Chemical Society, copyright 2008”. (b). Illustration of an automatic sample collection-to-detection biosensor system for *Mycobacterium tuberculosis.*^202^ “Reproduced from ref. 202 with permission from The Royal Society of Chemistry, copyright 2022”. (c). Cotton-tipped electrochemical immunosensor for sampling to detection of SARS-CoV-2.^203^ “Reproduced from ref. 203 with permission from the American Chemical Society, copyright 2021”.

Automatic samplers offer a promising direction for enabling automatic sampling-to-detection applications, such as monitoring pathogen presence at specific locations. To establish an automated pathogen monitoring network in air, enabling automatic sampling of bioaerosols and converting them into liquid samples is a critical preparatory step. Conventional sampling methods include impaction, suction, electrostatic precipitation, filtration, and impingement (Fig. 8). In impaction methods, aerosol pathogens physically settle onto a solid surface, and adhesive-coated surfaces like petroleum jelly-coated surfaces are explored to enhance the passive impaction of aerosol pathogens.^194,195^ Although impaction is relatively inexpensive, it faces challenges in terms of efficiency and quantification. In contrast, impingement methods often use bubbling liquids to efficiently capture small pathogenic organisms and are more favoured in recent aerosol monitoring research.^196,197^ For example, researchers have employed liquid-phase samplers to collect airborne COVID-19 virus for subsequent PCR-based tests.^190,198^ However, challenges in impingement methods include small sample volumes and liquid loss due to evaporation. Filtration and suction methods can be combined, where a vacuum pump creates a pressure differential to accelerate airflow, and a filtration system collects pathogens of a specific size. This approach has been widely used with microfluidic channels to create an integrated sampling and detection system, although it suffers from lengthy operational processes such as transferring samples from a filter to microfluidic channels and culturing samples.^199^ The electrostatic precipitation methods utilize the natural charges of bacteria; *e.g.*, most bacteria have a negative charge at neutral pH and an electropositive filter can be used to enhance pathogen capture.^200^ Each method presents unique advantages and challenges for integration into automated pathogen monitoring systems, and continued research is needed to improve the efficiency and scalability of these technologies.

The real-time detection of infectious bioaerosols in the air with automated detection systems is particularly challenging due to several factors. First, the concentration of infectious pathogens in the air is extremely low, requiring highly efficient sampling methods to concentrate pathogens above the LOD for detection methods. Unfortunately, many available air samplers are not efficient enough at concentrating pathogens. Moreover, the loss of analytes due to the deterioration of aerosol pathogens during sampling—such as damage to antigen surface proteins or peptides—can significantly hinder detection capabilities. Factors like environmental stress, cell damage during sampling, and hydration all play important roles in the deterioration process of bio samples.^197,204^ Additionally, dust particles and biological contaminants like saliva or sputum in the air can interfere with sampler collection and biosensing methods. Dust particles, typically ranging from 1–100 nm in air, scatter light at different angles and intensities and can cause complications with optical-based biosensing methods, resulting in lowered sensitivity and specificity.^199^ Dust particles can also clog the air samplers, potentially leading to malfunction.^205^ To address these issues, parameters such as sampler location, filter size, sampling methods, and air flow rate need to be optimized in order to properly sample and concentrate the low-level pathogens in air for effective automated airborne bioaerosol detection.

An ideal integrated automatic biosensing platform should consist of both a highly efficient automated sampler and a highly sensitive portable diagnostic device. While there is extensive research on each component individually, studies focusing on the integration of these systems are comparatively fewer.^206^ The feasibility of integrating automatic samplers with various detection techniques has been reported. For example, in 2008, an Autonomous Pathogen Detection System (APDS) is first demonstrated and accepted by the US government (Fig. 8a), marking a significant advancement in this field. The APDS is capable of automatically collecting and selectively concentrating particles from the air into liquid samples, which are then analyzed using PCR-based methods within the system.^201^ The system utilizes a combination of filter screens and a wet-cyclone collector to convert airborne particles into liquid samples and has been successfully tested for detecting *Bacillus anthracis* and *Yersinia pestis* in both laboratories and high-traffic areas such as trains. Despite its potential, the overall size of the device is relatively bulky and its detection capability is affected by background noise from dust and other airborne particles. Lee *et al.* developed a lateral flow assay (LFA) based integrated system for bioaerosol sampling and monitoring, demonstrating a response to MS2 bacteriophage and avian influenza virus (AIV) H1N1 in a simulated environment.^207^ The system utilizes a porous glass fiber pad to capture pathogens from the inlet airflow, which are then flushed with buffers into the integrated LFA for detection. Near-infrared (NIR) emission is used as a signal to indicate the presence of pathogens and an LOD down to 10^6^ PFU mL^−1^ is achieved for MS2 virus, which is 10-fold lower than that of commercial kits.

With the continued development of microfluidic channels and lab-on-a-chip technologies, FET-based devices have become promising candidates for POC automated airborne pathogen detection due to their miniaturization capabilities. Handheld signal readers can be used to read FET-based biosensor signals, unlike the bulkier readers required for optical-and PCR-based detection methods. Although the FET-based biosensors hold huge potential in the automated pathogen detection system, relatively little research has been conducted on integrating microfluidic-based FET sensing platforms with aerosol samplers for pathogen detection and monitoring. Ma *et al.* developed a Si-nanowire based FET biosensor integrated with samplers to detect *Mycobacterium tuberculosis* from patients’ exhalations (Fig. 8b).^202^ The sensor system collected a sufficient sample volume in less than 2 minutes and could detect MTB proteins in sputum-equivalent breath condensate (SEBC) at concentrations as low as 4 × 10^4^ particles per mL. The packaged sensor is flexible and portable with a size of 36 mm by 31 mm. Similarly, Lee *et al.* designed a CNT-based FET that is able to detect two different kinds of airborne allergic fungal particle simultaneously.^208^ The CNT-based FET was integrated with a commercial air-to-liquid sampler through a predesigned microfluidic channel to achieve automated sampling and detection. The system successfully detected *Alternaria alternata* and *Aspergillus niger* in the range of 10–10^6^ pg mL^−1^. In addition, a Peltier cooler was incorporated to enhance the receptor stability, extending the sensor shelf-life up to 9 days.

Beyond the traditional approach of connecting an external sampler to the sensing device, sampling may be merged with sensing on the same chip, creating a self-diagnostic kit. Eissa *et al.* fabricated an electrochemical biosensor for COVID-19 detection using screen-printing technology (Fig. 8c). In this design, cotton fibers were incorporated onto the detection zone of the electrode to enable same-chip sampling and detection. The immunosensor was fabricated by immobilizing the virus antigen on CNF-modified screen-printed electrodes, and the detection of virus antigen was achieved *via* competitive assay. After sampling the nasal samples, the chip is immersed into PCR tubes containing a fixed amount of N protein antibodies in the solution; typically, a higher concentration of virus in the sample results in fewer antibodies binding to the sensor surface. The impact of cotton on the signal was found to be insignificant through comparison experiments.^203^ Considering the advancements in this field, integrating FET biosensors with samplers for automated detection holds great promise. The continued development of these technologies could lead to a new generation of POC biosensors with wide applications for real-time, automated pathogen detection.

### Integration with wearable devices

Wearable designs can be incorporated with transistor biosensors for everyday healthcare, assisting patients in monitoring critical biological entities. The popularity of wearable devices has surged with the advancement of smartphones and the IoT. To date, biosensors have been incorporated into a wide range of wearable devices, including clothing, bandages, rings, masks, tattoos, and contact lenses.^209^

Wearable devices can be classified into two categories: casual use and medical-care specific. Casual wearables, such as the Apple watch, are primarily designed to track physical activity and fitness, often serving as both a health tool and an entertainment device. On the other hand, medical-care-specific wearable devices are developed to monitor specific physiological parameters and provide critical health insights. These devices can be worn on the skin or specific locations on the body; for example, some continuous glucose monitors require the insertion of a sensor pellet under the skin of upper arm. Medical care-type wearable devices, such as glucose monitors, provide continuous monitoring of specific physiological activities on a hourly basis, daily basis, or weekly basis and may require replacement after certain periods of time.^210^

Key considerations for the development of excellent wearable devices include miniaturization, efficient sampling methods, biocompatibility, flexibility, and reliable performance under various bending conditions. Currently studies on wearable devices are largely focused on non-invasive, dynamic monitoring of health through biofluids such as interstitial fluid, saliva, tear, and sweat.^209^ The applications of wearable devices require them to be lightweight and user-friendly to ensure ease of use and acceptance by end-users.

**Fig. 9:**
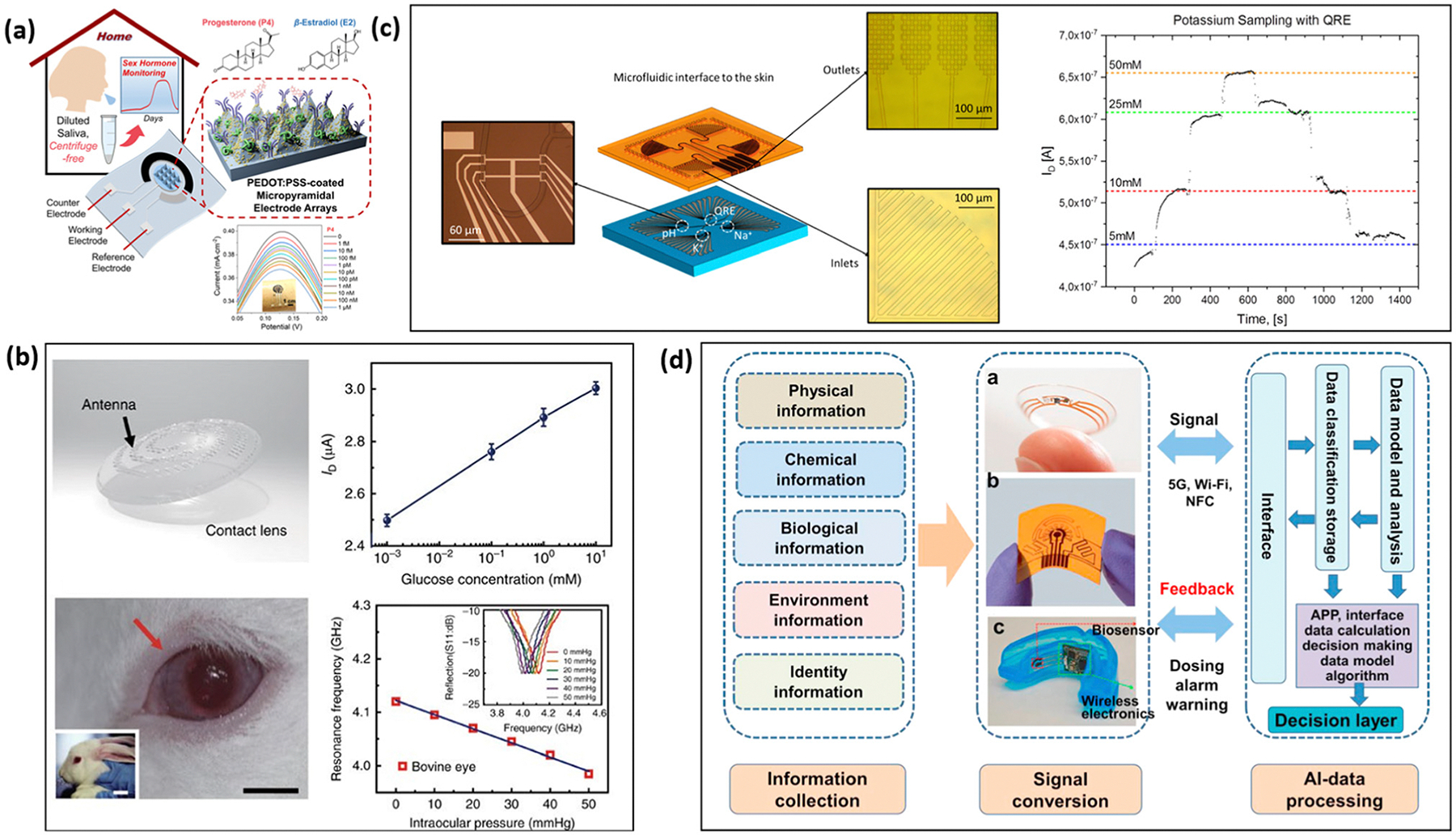
(a). Tip-enhanced sub-femtomolar salivary immunosensor for steroid sex hormones.^216^ “Reproduced from ref. 216 with permission from the American Chemical Society, copyright 2023”. (b). Wearable smart sensor system integrated on soft contact lenses for wireless ocular diagnostics.^217^ “Reproduced from ref. 217 with permission from Springer Nature, Creative Commons Attribution (CC BY) license, copyright 2017”. (c). Ultra-low-volume lab-on-skin passive microfluidics embedded ion-sensitive field-effect transistor for ion detection in sweat.^218^ “Reproduced from ref. 218 with permission from the American Chemical Society, copyright 2018”. (d). Schematics of potential biosensor integration with the IoT.^219^ “Reproduced from ref. 219 with permission from Elsevier, copyright 2020”.

Saliva is an oral biofluid containing a variety of components such as metabolites, hormones, enzymes, microorganisms, proteins, and ions that permeate from blood *via* transcellular or paracellular paths.^211^ Since saliva is more readily available compared with blood, it is an excellent non-invasive medium for monitoring the emotional, hormonal, nutritional, and metabolic state of the human body.^212^ Saliva can be sampled onto the sensing device either through passive drooling or with the aid of a swab.^213,214^ Klinghammer *et al.* demonstrated a multiplexed platform comprising an array of SiNW FET sensors for label-free monitoring of the stress biomarker cortisol in saliva.^215^ The sensor employed an aptamer probe for the detection and utilized the folding of the aptamer upon binding, close to the sensor surface, to overcome the Debye length limitations. Additionally, Li *et al.* developed a PEDOT-based electrochemical biosensor for at-home, centrifuge-free testing of the female ovarian hormone cycle, specifically detecting both progesterone (P4) and estradiol (E2) (Fig. 9a). The 3D micropyramidal arrays fabricated on the PEDOT surface were reported to significantly enhance the sensitivity of the sensor, achieving detection at sub-femtomolar (sub-fM) levels.^216^

Tears are a complex extracellular fluid consisting of proteins, electrolytes, peptides, lipids, and metabolites from various sources including the lacrimal glands, goblet cells, epithelial cells and blood.^209^ Since blood is one of the contributors to tears, tears can be used to monitor the glucose levels in diabetic patients. However, two main challenges hinder tear-based detection. First, in bench-top detection, the transportation of the tear sample to the lab can lead to evaporation which considerably affects the detection accuracy. Second, sampling tears from delicate human eyes poses a significant challenge and requires extreme caution. Given these challenges, smart contact lenses have emerged as a promising candidate for real-time monitoring of parameters such as glucose level, intraocular pressure, and pH.^220^ Park *et al.* demonstrated FET-based soft contact lenses for glucose monitoring, using pyrene linker-aided glucose oxidase probes immobilized on the graphene surface (Fig. 9b).^217^ The soft contact lens has been tested both *in vivo* and *in vitro* using live rabbit and bovine eyeballs, demonstrating reliable operation with an LOD down to 0.4 × 10^−6^ M.

Up to 30% of bodily waste is secreted through perspiration from the sweat glands in the human skin during daily exercise. Sweat contains a variety of chemicals, including ions, metabolites, acids, hormones, small proteins, and peptides, offering abundant information on people’s health status.^221^ Given its rich composition, sweat can be used to monitor physiological conditions such as intoxication levels and drug abuse.^222,223^ With the recent advancements in wearable autonomous collection platforms, it is possible to achieve non-invasive collection and real-time analysis of sweat directly from the skin. Wang *et al.* demonstrated a wearable non-invasive biosensor for continuous monitoring of essential amino acids and vitamins in sweat during exercise and at rest.^224^ The incorporation of carbachol hydrogel at the anode and cathode facilitates efficient microfluidic-based sweat sampling, enabling autonomous and continuous sweat monitoring with high accuracy across activities including physical exercise and at rest.

Skin interstitial fluid (ISF), which surrounds cells and can be collected through diffusion, plays a crucial role in delivering nutrients and removing waste *via* the blood capillaries. This fluid contains a wealth of vital information, making it an excellent medium for continuous health assessments. Detection of glucose levels through interstitial fluid has been an extensive focus over the past several decades. Various biosensors based on Raman spectroscopy, photoacoustic spectroscopy, electro-magnetic waves, and FET have been developed for glucose monitoring.^225^ Some ISF biosensors utilize microneedles for sampling,^226,227^ while many commercial glucose sensors utilize non-invasive iontophoresis-based techniques, such as the GlucoWatch^®^, GlucoTrack^™^, G2 Biographer device and OrSense NBM-200G,^209,228^ offering a more convenient and less painful alternative for patients.

One important integration for wearable devices is the incorporation of microfluidic channels for automated, passive biofluid collection. Zou *et al.* demonstrated a microfluidic channel integrated extended-gate FET-based sensor for pH detection.^181^ The fluid inlet in the microfluidic channel is driven by a capillary flow pump, enabling body fluid collection during wearing. Additionally, a temperature detection pad was incorporated into the sensor for temperature compensation, with measurement results showing a deviation of only 0.17 °C from the gold standard. The microfluidic channel can be fabricated cost-effectively using 3D printing, achieving a 200 μm accuracy with a printing time of less than 10 minutes, high lighting the potential for mass production. Similarly, Garcia-Cordero *et al.* developed an ion-sensitive lab-on-skin biosensor which can passively collect sweat from a person’s skin *via* capillary forces, allowing for the detection of pH levels and concentrations of Na^+^ and K^+^ ions (Fig. 9c).^218^

Compared with traditional POC sensors, wearable devices are designed to be worn on the wrist or other body parts, requiring flexibility to fit closely on various skin surfaces for efficient biofluid collection. This translates into a unique demand for wearable sensors that traditional FET-based POC devices do not face: high endurance toward bending cycles and the ability to maintain consistent performance under various curvatures. E. Laliberte *et al.* developed a wearable graphene-based FET on 50 nm SiO_2_-coated flexible Kapton film that addresses these challenges. The wearable device achieves a detection range of 10 pM to 100 nM for interleukin-6 (IL-6) protein, a key biomarker in immune responses. The wearable biosensors can withstand a 1.5 cm to 4.25 cm radius of curvature while maintaining proper functionality. The wearable biosensor is also integrated with a microfluidic channel and a specially designed portable signal meter, allowing for real-time, dynamic monitoring of IL-6 with portable readouts.^229^ While organic soft materials are often preferred for their flexibility, research has shown that inorganic materials such as graphene and indium oxide can also be used to fabricate highly flexible biosensors with minimal performance loss during bending cycles.^230^ For example, Chen *et al.* demonstrated polymer-based OECT that maintained stable electrochemical properties over 1500 redox cycles under 10^4^ stretching cycles of 30% strain.^231^

Wearable devices, as their name implies, require direct contact with the skin, necessitating the use of biocompatible materials to ensure the safety and comfort of the wearer. In the future, wearable devices are expected to evolve into implantable sensors for more precise monitoring, especially for patients with high-risk conditions. In terms of biocompatibility, most metal-based materials such as Ag, Cu, and Al are not ideal for biosensors as these metals can penetrate body tissue after prolonged skin contact, posing potential health risks^232^ In contrast, inert metals like gold (Au), platinum (Pt), and titanium (Ti), along with carbon-based and ceramic composite materials, offer better biocompatibility and are potential candidates for wearable biosensors. Organic materials, however, provide an even broader range of options, such as PEDOT:PSS and hydrogel materials, offering the flexibility to meet specific functional requirements. Lots of different OECTs have been developed for wearable applications. For example, J. Currano *et al.* developed a PEDOT:PSS-based sensor pad on a Kapton substrate that can successfully detect lactate in sweat, exhibiting an electrical sensitivity of 1.9 mA mM^−1^.^233^ Fumeaux *et al.* designed a disposable, biocompatible OECT made from polylactic acid (PLA), carbon, PEDOT:PSS, and PMMA. The device has carbon and PEDOT:PSS printed on top of the PLA substrate as electrodes and gate, respectively, and PMMA-based microfluidic channels fabricated on top of the devices. The sensor demonstrated linear detection for glucose up to 1 mM, showcasing the potential of these materials for wearable, non-invasive health monitoring.^234^

Several challenges remain in the translation of wearable devices for real-world applications, including standardization of the fabrication process, ensuring device stretchability, efficient sample detection, and extending shelf life.^235^ The standardization of the fabrication process has been a long-standing issue for nearly all transistor-based biosensors, not just wearable devices. Current issues such as device-to-device variation, low device yield, and inconsistent surface functionalization fall short of meeting commercial requirements. In terms of stretchability, while numerous studies have demonstrated flexible wearable biosensors that can maintain performance under laboratory bending tests, real-world applications are far more complex, involving forces such as buckling, shear, and compression, which affect device durability and functionality. Similarly, while many laboratory sensor devices have shown the ability to automatically collect biofluids like sweat, real-life conditions introduce additional complications such as dirt, dust, and small particles that can clog microfluidic channels, potentially impairing functionality. Shelf life presents another significant challenge for wearable devices. Depending on the specific applications, users’ tolerance for the price and device longevity varies. For example, patients with diabetes might be willing to pay for a wearable device that needs to be replaced every one or two weeks if it can accurately detect glucose levels, as it is crucial for them. However, for most people looking to monitor their health for general wellness purposes, a device with less than one month of shelf life may not be an appealing choice for them. To enable the real-life translation of transistor-based wearable devices, extensive testing and optimization are required to overcome these challenges.

### Integration with the Internet of Things (IoT)

Sensors can be further integrated with smartphones or the IoT to enable wireless communication, extending their functionality to even form large sensor networks capable of acting as an “electronic nose” for automatically monitoring harmful molecules and pathogens over large areas. In the past decades, with the rapid development of artificial intelligence (AI), the IoT has become a powerful technology that seamlessly integrates with various electronic devices to support everyday life. The intelligent processing of biosensing data is revolutionary for applications in healthcare and public health. In the realm of public health, AI-integrated biosensors can establish large areas of sensor networks to monitor the outbreak or transmission of certain diseases, allowing governments and health authorities to take timely control. In personal healthcare, AI-integrated biosensors can enable one medical team to monitor the health conditions of a large number of patients, allowing for prompt interventions when abnormal health patterns are detected. Furthermore, individuals could remotely monitor the health of elderly family members or patients with chronic diseases, offering increased flexibility and convenience in caregiving. There is no doubt that the future of biosensing will be shaped by the integration of smart technologies—ushering in an era of intelligence.

The basic architecture of AI-biosensors is composed of three main elements: information collector, signal transducer, and cloud data processing (Fig. 9d).^219^ The information collector refers to the integration of biosensors with samplers, microfluidic channels, or wearable devices. These integrations enable the collection of target biomolecules from various sources, such as body fluids or environmental samples. The signal transducer refers to the component of the biosensor responsible for converting the presence or concentration of biomolecules into electrical signals. The last part, cloud data processing, refers to the processing and analysis of electrical signals with AI techniques. The data undergoes classification, modelling, and analysis, allowing for advanced pattern recognition and decision-making.

The three-part architecture offers a comprehensive framework for understanding the biosensor translation process. The signal transducer part represents the development of biosensors in the laboratory, where the primary focus is on optimizing the performance of the sensor itself. Key objectives in this phase include achieving a lower limit of detection, expanding the sensing range, enhancing selectivity, and simplifying the sensing process. This phase defines the technical capabilities of the biosensor. The information collector part can be analogous to the front end of the biosensor system, where the ultimate goal is to develop innovative methods to automatically collect and process the sample in real-time. This stage ensures that the biosensor can seamlessly interact with the environment to effectively capture analytes with minimal human intervention. The last, cloud data-processing, part is the step for the translation of biosensors into real healthcare solutions. Biosensors equipped with integrated information collectors alone are considered as semi-finished products. It is only through further integration with cloud data analysis that biosensors can evolve into next-generation healthcare tools.

Given the popularity of smartphones in this era, the integration of smartphone technology for biosensor actuation and monitoring has garnered significant attention. Smartphones can be directly or wirelessly connected to electronic analyzers and sensor chips for the detection of specific biomolecules, offering a convenient and user-friendly platform for real-time monitoring. For example, Zhao *et al.* has developed an ultra-sensitive electrochemical sensor targeting the RNA of SARS-CoV-2 using calixarene-functionalized graphene oxide. The device eliminates the need for specific nucleic acid amplification and is directly connected to smartphones.^236^ Similarly, Su *et al.* demonstrated the use of smartphones as wireless data acceptors for the detection of serum-derived exosomes and secretion monitoring.^237^

Another emerging field is the integration of machine learning (ML) with biosensors. ML enhances data analysis by extracting more meaningful information from raw sensor data. Its applications include: (1) categorizing the sensing signal into various groups through algorithms based on different analytes, (2) reducing background noise during detection, such as sensor internal drifts, contaminations, and environmental fluctuations, (3) ruling out abnormal signals to improve the accuracy of detection, and (4) identifying patterns and signals that might be overlooked by human analysis.^238^ For example, using ML trained from multi-dimensional information collected through multi-sensor arrays, Xue *et al.* demonstrated improved system functionality and accuracy for multipleion detection in complex solutions.^239^ By employing a random forest algorithm, their system accurately classified ion types, predicted ion concentrations, and analyzed electrolyte imbalances related to diseases.^226^

Another key integration involves wireless data transmission and the formation of sensor networks. This idea is similar to the real-time continuous soil monitoring networks.^240^ The integration of wireless data transmission, sensor network, and AI analysis represents the next frontier for biosensors in real-life applications. Biosensors could form vast sensor networks where each node is a portable biosensor that autonomously samples and detects bioaerosols in the air. Data from these nodes can be wirelessly transmitted to the cloud for machine learning analysis, facilitating timely responses by governments. Biosensors can also be deployed in retirement communities and disseminated to each resident to help doctors quickly respond to emergencies experienced by residents. As biosensing and AI technologies continue to evolve, the applications of these integrated platforms will expand significantly, broadening their impact on public health and personalized healthcare.

## 4. Conclusions and outlook

Biosensors are expected to become a central focus in the coming decades due to their close connection to public and personal health. Transistor-based biosensors are especially promising candidates among the various types of biosensor, given their portability, sensitivity, and accuracy. Additive manufacturing presents a significant advantage for the mass fabrication of transistor biosensors with minimum sample load, drop-on-demand deposition, and customizability. However, several challenges remain, such as the need for higher printing precision and potential clogging of the nozzle, which affects almost all printing methods. Compared with conventional fabrication techniques like CVD and drop casting, additive manufacturing acts as a balanced choice for depositing nanomaterials—producing a high-quality film with a controllable thickness both time- and cost-efficiently. It is anticipated that advanced printing techniques will become important fabrication methods in the future for not only laboratory production but also the mass production of high-quality transistor-based biosensors. The development of 3D printing technology may enable onsite sensor production according to demand, which would be extremely valued in remote rural area or countries with limited transportation and harsh storage environments. The ability to fabricate sensors onsite based on immediate needs also addresses concerns about sensor shelf-life and transportation logistics.

For promising laboratory-developed devices, it is important to translate them into real-world applications. The first step of translation is the integration with lab-on-a-chip and microfluidic platforms. The lab-on-a-chip integration prepares the biosensors for mass-production, while also enhancing the portability of biosensors for various applications. Integration with microfluidic platforms enables the detection of various biomolecules in dynamic environments, such as continuous flow systems, and builds the cornerstone for further functionality. Some of these further functionalities could include the integration of samplers to improve information collection such as autonomous samplers for large-area monitoring and wearable devices for personal healthcare. Another further functionality is improving the wireless communication and data analysis ability through integration with the IoT. Cost-effective wireless sensors could also be deployed in networks to remotely monitor the presence of hazardous bioaerosols in large areas and help governments to take timely control of any outbreak of epidemic.

For successful commercialization of transistor-based biosensors, it is essential for these sensors to differentiate themselves not only through performance—sensitivity and selectivity—but also through device integrations, such as wearability, IoT capabilities, and user interfaces (UIs). As more research begins to focus on the functional integration of devices for transistor-based biosensors, alongside advances in nanomaterials and structures, the translation of these biosensors into practical applications is expected to accelerate in the coming years. Despite research being conducted over the past decades on transistor-based biosensors, this type of sensor is still considered relatively new to the market. Regulatory approvals and clinical validations present significant hurdles that must be overcome before translating these technologies into commercial products. Regulatory agencies classify biosensors as medical devices, necessitating stringent validation before they can be marketed. Transistor-based biosensors for POC diagnostics, disease detection and biomarker monitoring fall under moderate to high-risk categories, demanding extensive tests and approval processes. As emerging technologies, transistor-based biosensors face the challenge of establishing global regulatory standards and integrating with existing diagnostic standards. Clinical validation is essential for demonstrating the effectiveness of these biosensors in complex real-world diagnostic environments, typically involving multiple testing phases, such as laboratory-based analytical validation, preclinical testing, and clinical trials. This entire validation process can take years to complete, which is why transistor-based biosensors are still progressing toward commercialization. While few FET-based sensors have been commercialized, Sentron has successfully introduced an ion-sensitive field-effect transistor (ISFET) for pH detection.^241^ Similarly, Paragraf has commercialized a graphene FET for both potassium and pH detection.^242^ A notable FET biosensor nearing commercialization is being developed by QuLab Medical, a medical device and digital health startup, which announced in 2023 a new multi-metabolite wearable sensor for type I diabetes (T1D) management based on its patented FET sensors.^243,244^ Despite these challenges, transistor-based biosensors, with their portability, sensitivity, and potential for integration, will likely play an important role in the next era in fields such as personalized healthcare, environmental monitoring, smart cities, and infrastructure management, as challenges related to shelf life, yield, cost, regulatory approvals, and clinical validations continue to be addressed.

## Figures and Tables

**Table 1:** Comparison of key aspects for screen, inkjet, and aerosol-jet printing, digital light processing technologies, spin coating and chemical vapor deposition in the fabrication of transistor-based biosensors

Fabrication technique	One-step fabrication	Process	Materials	Challenges	Advantages

Screen printing	✓	Fill mesh with ink and lift off	Metal nanoparticles, conducting polymers	Yield, clog of nozzle, reliability	Versatility, mass production, low cost, Line width: 30–70 μm
IJP	✓	Droplet by droplet through thermal or piezoelectric force	0–2D semiconductors, metal nanoparticles, conducting polymers	Clog of nozzle, coffee ring effect, ink development	Drop on demand, contactless printing, rapid prototyping, line width: 20–30 μm
AJP	✓	Atomize and print with micro-scale droplet	0–2D semiconductors, metal nanoparticles, conducting polymers	Clog of nozzle, printing speed, reproducibility	Drop on demand, contactless printing, line width: 10–120 μm
DLP	✓	Photopolymerization	Conducting polymers	Limit to photosensitive polymers	3D printing, high throughput, contactless printing, line width: 3–100 μm
Spin Coating	×	Spin liquid material to form a film with desired thickness	0–2D semiconductors, conducting polymers	Thickness control, edge effects, surface compatibility, film quality	Simplicity and cost-effectiveness, rapid prototyping, scalability
CVD	×	Through chemical reactions of gaseous precursors	0–2D semiconductors, metal oxides	Process complexity, uniformity, scalability, high temperature and vacuum conditions, time efforts	High-quality deposition, precision and control, high-performance sensor
Conventional lithography	√	Mask-based multi-step lithography	Silicon-based semiconductor, metals, metal oxides	Limited to rigid silicon wafers, cleanroom needed	Sub-10 nm feature sizes, high performance and uniformity, fully industrialized for scalable production

## Data Availability

No primary research results, software or code have been included and no new data were generated or analysed as part of this review.
